# The Effectiveness of Dengue Vector Control: A Meta‐Review

**DOI:** 10.1111/tmi.70018

**Published:** 2025-08-21

**Authors:** Gary K. K. Low, Sam Froze Jiee, Siong Hee Lim, Osamudiamen Favour Omosumwen, Selvanaayagam Shanmuganathan

**Affiliations:** ^1^ Sydney Medical School, Faculty of Medicine and Health University of Sydney Sydney New South Wales Australia; ^2^ Research Directorate Nepean Hospital, Nepean Blue Mountain Local Health District Kingswood New South Wales Australia; ^3^ Department of Community Medicine and Public Health, Faculty of Medicine and Health Sciences Universiti Malaysia Sarawak Kota Samarahan Sarawak Malaysia; ^4^ Alberta Health Service Edmonton Alberta Canada; ^5^ Menzies Centre for Health Policy and Economics, Faculty of Medicine and Health The University of Sydney Sydney New South Wales Australia; ^6^ Ministry of Health Putrajaya Malaysia

**Keywords:** *Aedes*, dengue, mosquito, prevention, vector

## Abstract

**Background:**

Dengue vector control plays an important role in reducing the burden of dengue infection. This study aimed to summarise the evidence of published systematic reviews on the efficacy of dengue vector control interventions.

**Methods:**

Systematic reviews of cluster randomised controlled trials and randomised controlled trials in populations/people exposed to the risk of dengue infection in the presence of the vector were included. All dengue vector control, all comparators and any outcomes were considered in this review. Electronic databases and reference lists were searched. Screening, full‐text reviews, data extractions and quality assessments were conducted independently by two reviewers with resolution by a third reviewer.

**Results:**

A total of 15 systematic reviews were included in this study, but narrative synthesis was performed for only 3 reviews that reviewed cluster randomised controlled trials or randomised controlled trials. Community mobilisation and insecticide‐treated materials were weakly effective interventions reported by two systematic reviews that have acceptable methodological quality. However, the non‐overlapping of randomised controlled trials and cluster randomised controlled trials included in their respective reviews may affect the findings.

**Conclusion:**

There is insufficient evidence to recommend a method of dengue vector control management. Novel dengue vector control methods are highly encouraged for urgent trials. Until then, the current respective local governments' vector control management may still play a vital role in controlling the mosquito's propagation and transmission of dengue infection.

## 1 Introduction

The World Health Organization (WHO) documented over five million cases of dengue infection across 129 countries in 2019, with 5000 reported dengue‐related deaths in early 2023 [1]. Dengue fever is transmitted by a mosquito vector, *Aedes aegypti*, which is also the vector for yellow fever, chikungunya and Zika viruses [2]. Therefore, dengue vector control plays an important role in reducing the burden of dengue infection.

The World Health Assembly called on its Member States to develop or adapt national vector control strategies and operational plans. As a result of this, resolution WHA 70.16: an integrated approach for the control of vector‐borne diseases was adopted in May 2017 [3]. The goal of this strategy is to reduce the case incidence and mortality due to vector‐borne diseases by 2030. The WHO guideline recommends employing locally adapted sustainable vector controls either alone or in combination with environmental management, chemical control, individual and household protection and biological control [4].

Systematic reviews and meta‐analyses on the effectiveness of dengue vector controls have been useful in informing public health practitioners on the choice of suitable vector controls. However, the recommendations of these systematic reviews are too specific to a geographical location [5, 6], only focused on household‐level protection [7] or on educational messages [8]. Furthermore, high‐quality systematic reviews that examined randomised controlled trials (RCTs) have yielded varying findings and conclusions [9, 10, 11]. The different findings among the systematic reviews make it challenging to be adopted for dengue vector control implementation [12]. Public health practitioners need timely information for rapid disease control. Hence, this study aimed to summarise the evidence of published systematic reviews on the efficacy of dengue vector control interventions.

## 2 Methods

This meta‐review (overview of systematic review) was conducted in accordance with the Cochrane method for overview of systematic reviews [13]. We have followed the Preferred Reporting Items for Systematic Reviews and Meta‐Analyses (PRISMA) 2020 checklist for this review [14]. This study was registered in OSF registry: https://osf.io/g5qxt.

### 2.1 Criteria for Considering Reviews for Inclusion

We have included systematic reviews of cluster randomised controlled trials (cRCTs) and RCTs, with or without meta‐analysis. Their methodological quality has been assessed in our review. For a better‐quality trial design, we decided to include reviews that reported results of RCTs or cRCTs. The participants in the included reviews were population/people exposed to the risk of dengue infection in the presence of the vector. All interventions on dengue vector control (prevention) and all comparators were considered in this review. Outcomes measured for the included reviews considered were entomological indices such as the Breteau index (BI), house, and pupae per person (PPI), household index (HI), container index (CI), tank positivity, number of mosquito adults; dengue incidence (any reported case data, clinical, lab‐confirmed or serologically positive cases); and any other outcome measures reported in the eligible systematic review were also included.

#### 2.1.1 Search Methods for Identification of Reviews

An electronic search was performed using PubMed, OVID (all EBM Reviews) and Cochrane Library. The search was performed on 2nd May 2024. No language restriction or date of publication was imposed. An additional search through the reference list of included reviews was performed.

The keywords ‘vecto’, ‘contro’ and ‘mosquit’ were combined with the Boolean operator OR, which was then combined with ‘dengue,’ using Boolean operator AND. Since the MeSH term of ‘mosquit’ includes ‘*Aede*’, we omitted this term in our Boolean combinations. A filter was employed to limit the search to only ‘systematic review’ and ‘meta‐analysis’ in PubMed; ‘human’, ‘systematic review’ and removal of preprint records in OVID; and limited to only ‘Cochrane Reviews’ in the Cochrane Library. Additionally, due to the default setting of OVID search for all EBM Reviews, we manually removed publications derived from Cochrane Clinical Answers, Cochrane Central Register of Controlled Trials and Cochrane Methodology Register.

### 2.2 Data Collection and Analysis

#### 2.2.1 Selection of Reviews

Duplicates were removed by matching the DOIs and titles using R version 4.4.0. Additional duplicates identified during the screening process were removed manually. Titles were then screened independently by two reviewers. Any disagreement was resolved by a third reviewer. Subsequently, full texts were retrieved for review independently by the two reviewers with a resolution by a third reviewer if there was disagreement.

### 2.3 Data Extraction and Management

A pre‐piloted data extraction form was used to extract the data. The data were extracted and cross‐checked for any discrepancies by the independent reviewers. The discrepancies were resolved by a third reviewer.

### 2.4 Assessment of Methodological Quality of Included Reviews

The AMSTAR 2: a critical appraisal tool for systematic reviews that include randomised or non‐randomised studies of healthcare interventions, was used to assess the quality of the reviews by two independent reviewers, with a third reviewer to resolve any discrepancies [15]. Additionally, the percentage of scores was calculated for each of the reviews by the sum of ‘yes’ as 1 and ‘partial yes’ as 0.5, over a total of applicable questions.

### 2.5 Data Synthesis

A narrative synthesis was performed along with a table to summarise each of the systematic reviews' characteristics, a summary of the quality of evidence within individual systematic reviews, and a conclusion of the individual reviews. We were unable to isolate and assess specific conclusions on RCT and cRCT in 12 studies with mixed primary studies. These were narrated in Section 3, but Sections 4 and 5 were based on systematic reviews that only included RCTs and cRCTs to ensure the quality of this meta‐review. No quantitative data analysis was conducted. R version 4.4.0 and package ccaR were used for calculating the Corrected Covered Area (CCA) % to assess the degree of overlapping primary studies among the included systematic reviews [16, 17]. High overlap can introduce bias in the synthesis by overrepresenting certain studies, while low overlap suggests more independent evidence sources. CCA % values lower than five indicate slight overlap and values greater than or equal to 15 indicate high overlap. CCA % was calculated by the formula:

$$\mathrm{CCA}=\frac{N-r}{\left(r\times c\right)-r}$$

where *N* is the total number of included publications (including double counting) in evidence synthesis; *r* is the number of rows (number of index publications); and *c* is the number of columns (number of reviews). CCA is then expressed as a percentage.

## 3 Results

**FIGURE 1:**
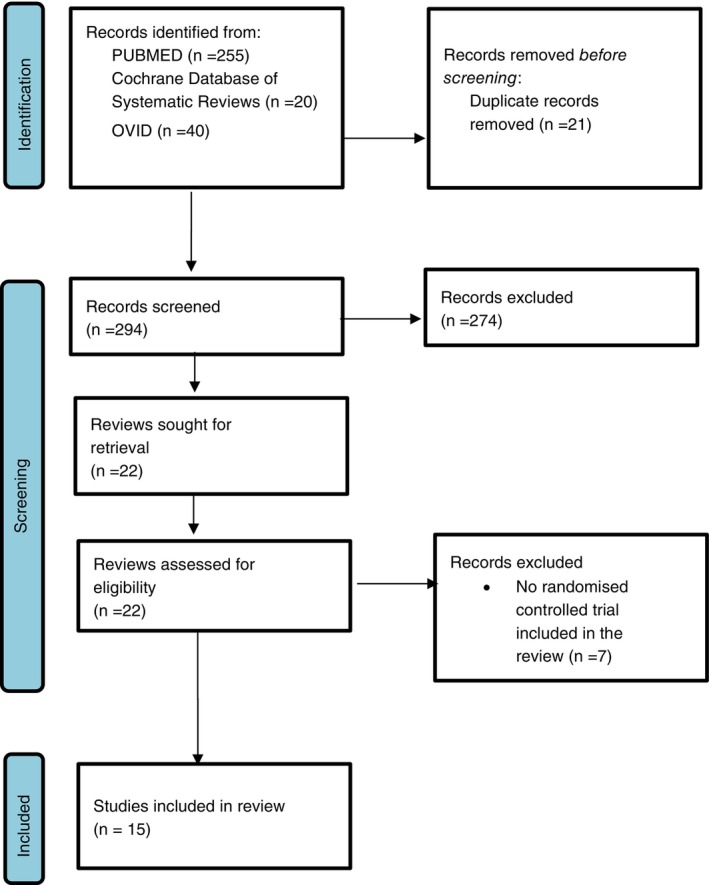
PRISMA 2020 flow diagram.

A total of 15 systematic reviews were included in this study. A PRISMA 2020 flow diagram of the article retrieval process and reasons for exclusion is provided in Figure 1. The excluded studies after full‐text review are listed in Table S1.

Among the 15 studies, three systematic reviews focused solely on RCTs and cRCTs alone [10, 11, 18], and 12 included other types of study designs as well, such as non‐RCTs, quasi‐experimental and observational studies [5, 7, 9, 19, 20, 21, 22, 23, 24, 25, 26, 27]. The studies were published between 2009 and 2023. Among the three studies that reviewed only cRCTs and RCTs, two assessed the quality of the included articles using the Cochrane risk of bias tool, and one used the Consolidated Standards of Reporting Trials (CONSORT) to evaluate the adequacy of reporting. Among the 12 that have mixed designs in their reviews, four used the Cochrane tool, two used CONSORT, two did not mention what was used to assess the quality and four did not assess at all. The study characteristics are tabulated in Table 1.

**TABLE 1:** The study characteristics of included systematic reviews (condensed version, see Table S2 for expanded version).

Study ID	Review title	Date of search	No. studies included (no. clusters, households or countries in included studies)	Inclusion criteria	Comparison interventions	Outcomes for which data were reported that could be included in an analysis	Summary of quality of evidence in reviews (risk of bias)
Alvarado‐Castro 2017	Assessing the effects of interventions for *Aedes aegypti* control: systematic review and meta‐analysis of cluster randomised controlled trials	January 2003 and June 2013 (updated the search in November 2016 to cover articles published up to the end of October 2016)	18 studies (246 intervention clusters, 48,131 intervention households) and 288 control clusters (69,430 control households) in 13 countries	Studies concerned directly with the impact of chemical control, biological control or community mobilisation, alone or in combination, on dengue vector parameters; studies that were cRCTs; and studies that provided information about at least one of the three standard *Ae. aegypti* indices: HI—households with larvae or pupae as a proportion of households examined; CI—containers with larvae or pupae as a proportion of containers examined; and BI—containers with larvae or pupae as a proportion of households examined	Chemical control interventions 1% temephos applied to household's water containers 3 monthly. Community removal of ‘removable’ water containers versus community removal of ‘removable’ water containers Window curtains with lambdacyhalothrin and pyriproxyfen chips in water (households took out chips; not considered further) versus control clusters received no interventions Insecticide (permethrin) treated bednets (ITNs) supplied to households versus no treatment for 5 months; received ITNs after 6 months … (see Table S2 for more details)	BI, CI measured monthly for 10 m in 300 households randomly selected in both intervention and control clusters HI, CI, BI and PPI measured at baseline, 4 weeks, 4, 12 (Mexico) and 9 m (Venezuela). Adult dengue IgM serology at baseline and 8 m in Venezuela in approx 650 households … (see Table S2 for more details)	Cochrane approach: six studies in the meta‐analysis as having a ‘low risk of bias’ and four as having an ‘unclear risk of bias’ Blinding of participants and personnel: 18 low risk of bias Blinding of outcome assessment: 17 unclear risk of bias, 1 low risk of bias Incomplete outcome data: 10 unclear risk of bias, 8 low risk of bias Selective reporting: 3 unclear risk of bias, 15 low risk of bias Other sources of bias: 18 unclear risk of bias
Buhler 2019	Environmental methods for dengue vector control—A systematic review and meta‐analysis	The literature search was performed until 30th May 2017	19 for qualitative synthesis and 16 for meta‐analysis Except one RCT, all studies were designed as cRCTs Geographically, most studies were conducted in South America (42%) and Central America (42%), with the remainder in South Asia (18%) and Southeast Asia (21%) … (see Table S2 for more details)	The eligibility criteria for the assessed literature were: (i) randomised controlled trials (RCTs) or cluster randomised controlled trials (cRCTs) with the following environmental vector control methods: (a) container covers without insecticides, (b) container covers with insecticides, (c) waste management with direct garbage collection, (d) waste management without direct garbage collection and (e) elimination of breeding places, (ii) outcome measures for pupal or larval indices, (iii) field studies conducted where dengue vectors naturally occur	Twelve studies combined two or more vector control methods. Container covers without insecticides were implemented in eight studies, more specifically all these studies used lids or nets on water storage containers. In six studies, container covers were implemented in parallel with waste management campaigns Four studies installed container covers with insecticides. Kroeger et al. applied lambdacyhalothrin to curtains in Mexico, whereas the other study by Kroeger et al. in Venezuela used firstly deltamethrin and after 5 months additionally lambdacyhalothrin was applied. Two studies treated water with Temephos and pyriproxyfen chips The waste management category was divided into two subcategories, with direct garbage collection (eight studies) and no garbage collection (five studies) … (see Table S2 for more details)	A total of 17 studies reported BI, 15 studies reported HI, 13 studies reported CI, 15 studies reported PPI and five studies reported the number of positive containers. Only the study by Overgaard et al. reported the number of mosquitoes per hour inside schools collected with a Prokopack aspirator. Three studies reported measures for human transmission as the number of pupil absence periods at school, cluster specific rates of dengue virus infection in paired saliva samples from children and the number of reported dengue cases and IgM serology	CONSORT on reporting structure. No overall conclusion
Horstick 2018	Protection of the house against Chagas disease, dengue, leishmaniasis, and lymphatic filariasis: a systematic review	1980–2017	12 out of 32 studies were dengue studies: Number of studies: 12 Number of households assessed: Between 187 and 18,838 Duration of follow‐up: Between 4 weeks and 24 months	Types of diseases included vector‐borne NTDs such as Chagas disease, dengue, leishmaniasis (both cutaneous and visceral) and lymphatic filariasis. Furthermore, vector control operations are applied in and around households. Types of studies were RCTs and cRCTs. The types of interventions included any intervention that aimed to reduce disease incidence through vector or … (see Table S2 for more details)	The combination of indoor malathion ultra‐low volume spraying with an educational campaign Insecticide‐treated curtains Insecticide‐treated curtains in open housing structures Insecticide treated‐screens in combination with Spinosad (a type of insecticide) Permanently mounted, insecticide treated‐screens fitted to the doors and windows of residential houses. Insecticide‐treated curtains (windows or doors) alone or in combination with insecticide‐treated water container covers … (see Table S2 for more details)	Larvae‐positive containers in households, PPI, HI, CI and BI, IgM seroprevalence (measured in a house‐to‐house serosurvey in Trujillo) adult density	Cochrane Collaboration's tool for assessing risk of bias and were scored Specifically for dengue control: 3 low quality was excluded 11 studies were deemed medium quality and 1 study as high quality
Ballenger‐Browning 2009	Multi‐modal *Ae. aegypti* mosquito reduction interventions and dengue fever prevention	Searches in PubMed, Science Direct, Google Scholar, and Cab Direct databases were conducted from July 2007 to January 2008	5 relevant studies (RCT/cRCT). RCT; *n* = 187 houses (45 controls) RCT; T1: *n* = 64 peridomestic drums 36 with routine water use, 18 with no water use (10 controls); T2: *n* = 28 tires (10 controls); T3: *n* = 68 flower vases at cemetery (23 controls) … (see Table S2 for more details)	Articles were included if an intervention was implemented and quantitative pre/post‐data on larval or adult indices was reported. Studies measuring mosquitoes other than *Ae. aegypti* or *Aedes albopictus* were excluded	T3: educational campaign only, C: no treatment; T1: malathion ULV spraying, T2: malathion spraying and educational campaign, C: no treatment T: door‐to‐door education campaign; C: no treatment T1: insecticide‐treated net curtains and pyriproxyfen in water containers, C1: untreated neighbourhood; T2: PermaNet insecticide‐treated curtains and covered water containers, C2: untreated neighbourhood T1: ovitrap with deltamethrin insecticide, C1: untreated neighbourhood, T2: same as T1, C2: same as C1 Biological: *Mesocyclops longisetus* copepods: T1: peridomestic drums 36 with routine water use, 18 with no water use. T2: tires. T3: flower vases at cemetery	BI and KAP CI BI, HI, PPI and serological testing BI and HI CI, Adult Density Index	Not done
Bardach 2019	Interventions for the control of *Ae. aegypti* in Latin America and the Caribbean: systematic review and meta‐analysis	January 2000 to September 2016	A total number of nine cluster randomised controlled trials (RCTs), of 15 relevant trials, could be meta‐analysed. (out of 75 included studies) 32 clusters consisting of 500 houses and 2000 inhabitants in Guantanamo Costarena city, two neighbourhoods City of Manaus. 12 clusters. 1487 houses Guantanamo.12 clusters (500 homes approximately) La Lisa, Havana. 16 intervention clusters (389 houses) … (see Table S2 for more details)	Experimental, quasi‐experimental and observational studies, economic assessments and qualitative studies related to control interventions on diseases transmitted by the *Ae. aegypti* mosquito, such as dengue, zika, chikungunya and yellow fever were considered. Studies conducted since 1995, assessing the control strategies Any epidemiological design, from LAC countries, reporting about the effectiveness or degree of implementation of vector control interventions of any kind The outcomes under consideration were: incidence and morbimortality of *Ae. aegypti* ‐related diseases, larval indices for … (see Table S2 for more details)	Biogents Sentineltraps (BGS) Adulticides and larvicides in the field Community engagement Insecticide‐treated bed nets and curtains Entomological surveillance as part of a control programme Insecticide‐treated bednets and curtains Entomological surveillance as part of a control programme Use of insecticides (larvae and adults) in the field Curtains and tulle screens soaked in insecticide Curtains and tulle screens soaked in insecticide Community engagement Insecticide‐treated bednets and curtains Community engagement—reduction in reservoirs Insecticides for indoor use, reduction in reservoirs, health team training	*Ae. aegypti* population density by questionnaire to inhabitants. Serological survey Mortality of adult mosquitoes BI, HI engagement, knowledge, perception and behaviour Number of positive containers per house Reservoir Index Infestation index Overdispersion index Specific dengue infection rate (saliva samples) in children aged 3–9 years CI, PPI	Cochrane Handbook: RCTs are of moderate or low methodological quality in most domains explored, except for the domain related to blinding of evaluators, where the risk of bias was generally low
Bowman 2016	Is dengue vector control deficient in effectiveness or evidence?: Systematic review and meta‐analysis	Publication dates: 1986 to 2014 Median year of publication: 2009	41 studies Study designs: RCTs: 9 (including 7 cRCTs and 2 RCTs) Non‐randomised studies: 32 Duration of studies: 5 months to 10 years Less than 1 year: 16 studies 1 to 3 years: 12 studies 8 or more years: 7 studies	Study design: Studies of any design published since 1980 were included Target vectors: Studies had to evaluate methods targeting *Ae. aegypti* or *Ae. albopictus* Duration: The studies needed to have a duration of at least 3 months Outcomes: Any study with empirical data reporting dengue incident data and/or entomological indices monitored longitudinally for the duration of the intervention. Dengue cases reported either by the study or obtained from external institutions (e.g., hospital records)	Community‐based advocacy, awareness CWGs, clean up, container covers, education (on use of larvicide), house inspections, water pipe repair Water tank covers CWGs for covering of water sources; not protecting artificial containers; not removing abate from drinking water Community‐based environmental management and water covers Insecticide‐treated curtains (×3) BGS traps Community participation to support existing campaigns	CI, HI, BI, PPI. Dengue incidence and dengue cases	Cochrane risk of bias tool for RCTs: Only 9 out of 41 studies were RCTs, indicating a limited representation of high‐quality evidence Most RCTs exhibited a high risk of bias, particularly due to inadequate blinding Selective outcome reporting was low risk in 9 studies, while incomplete outcome data had a low risk in 7 studies, with one study at medium risk and another at high risk … (see Table S2 for more details)
Boyce 2013	*B. thuringiensis israelensis* (Bti) for the control of dengue vectors: systematic literature review	The literature search and analysis was developed and carried out through March 2012	4 out of 14 with 3 cRCTs and 1 RCT 14 studies (368 interventional households, 4 interventional groups, 566 interventional containers, 31 intervention construction sites) and (92 control houses, 5 residential control areas, 227 control containers) in 8 countries	Research with an experimental design producing primary quantitative data Research conducted in the field, defined as any community or environment where dengue vectors naturally occur The use of Bti as a single agent to control dengue vectors Clear information on Bti formulation and dosing … (see Table S2 for more details)	Specific objective of the four studies: Characterise the resistance status of *Ae. aegypti* larvae from Martinique to conventional and alternative insecticides and to assess the efficacy and residual activity under simulated and field conditions Compare the efficacy of Abate (temephos) and Vectobac G against *Ae. albopictus* in bromeliads in the field Evaluate two control methods for *Ae. aegypti* that can be used by the community: lethal ovitraps and Bti briquettes Determine the influence of climate and of environmental vector control with or without insecticide on *Ae. aegypti* larval indices and pupal density	Larva density at 2, 4 and 6 weeks post intervention versus control group (without Bti intervention) Length of time until tank reinfested for treated versus untreated tank Length of time until tank reinfested for tank treated with different concentration of Bti. Hi, CI, OI measured at first, second and third month. HI, BI, CI measured and compare between interventional and control groups	CONSORT 2010 checklist: no risk of bias assessed
Esu 2010	Effectiveness of peridomestic space spraying with insecticide on dengue transmission; systematic review	Search conducted July 2008	1 cRCT study out of 15 studies included, from 10 countries. (1949 intervention household). (45 control household). 5 studies without control group. 6 studies did not clearly state the number of interventions household or cluster or containers involved 1 cRCT: Randomly selected houses drawn from approximately 7000 houses were assigned to 1 of 4 possible study arms … (see Table S2 for more details)	Peer‐reviewed publications that presented original data from field studies evaluating the effect of peridomestic space spraying on reducing wild *Aedes* vector populations and interrupting dengue transmission. RCTs, cRCTs, quasi‐randomised controlled trials, controlled before and after studies and interrupted time series studies. … (see Table S2 for more details)	Cluster randomised trial: Evaluation of an educational campaign for the elimination of *Ae. aegypti* breeding sites based on community participation, compared with ULV insecticide spraying (alone or in combination with community participation).	BI	No assessment conducted
George 2015	Community‐effectiveness of temephos for dengue vector control: A systematic literature review	Searches up to 15 June 2013	3 relevant studies (RCT/cRCT): Cluster RCT: 20 clusters (1835 houses); 18 months Randomised control trial experimental area: 17,994 houses (665 blocks); control area: 37,955 houses (1775 blocks); 10 months … (see Table S2 for more details)	Studies or programmes conducted with aim to prevent/control dengue Studies with quantitative outcomes such as BI, CI, HI, larval mortality indicated by pupal skins, average number of positive containers per house, pupal index, indoor resting density, ovitrap index or dengue incidence Community effectiveness studies … (see Table S2 for more details)	To test the efficacy, cost and feasibility of a combined approach of insecticide treated materials alone and in combination with targeted breeding site interventions To evaluate the efficacy of temephos for the control of the *Ae. aegypti* larvae To assess the effectiveness of an integrated community‐based environmental management strategy to control *Ae. aegypti*, compared with a routine strategy	HI, CI and BI were used to measure the outcomes. PPI and adult mosquito density were also measured Total production of *Aedes* pupae Pupae per person	No assessment conducted
Heintze 2017	What do community‐based dengue control programmes achieve? A systematic review of published evaluations	Up to March 2005	Number of studies: 11 (2 RCTs) Countries: The studies were conducted in various locations, with five studies in the Americas and six in Asia Households: The number of households targeted ranged from 5913 houses in one study to 100,000 individuals in another Clusters: Specific details on the number of clusters were not uniformly reported across all studies, but some studies involved multiple communes or villages	Original data: Publications must present original data from trials evaluating the effect of community‐based dengue control interventions Community‐based definition: The intervention must target the community, meaning at least one component should involve community participation (e.g., educational meetings, involvement of local leaders) Aim of the intervention: The primary aim should be to reduce the incidence of dengue disease or infestation of the community with *Aedes* mosquitoes, as measured by any entomological index Study design quality: … (see Table S2 for more details)	Exclusively community‐based dengue control Community‐based dengue control in combination with chemical larvicides	HI, BI, CI	The quality of the studies was scored on a scale from 0 to 8, with 8 indicating the highest quality 2 RCTs were rated as 7
Jaffal 2023	Current evidences of the efficacy of mosquito mass‐trapping interventions to reduce *Ae. aegypti* and *Ae. albopictus* populations and *Aedes*‐borne virus transmission	Searches were conducted on 25 February 2021	7 RCT out of 19 studies Intervention area: Group of 30 houses with LO control: A group of 30 houses without LOs. Both areas are located in the same neighbourhood: no buffer zone between intervention and control houses … (see Table S2 for more details)	Peer‐reviewed articles published before 25 February 2021 Target population: *Ae. albopictus* or *Ae. aegypti* Intervention: Use of lethal ovitraps or host‐seeking female traps to control mosquito populations Comparator: Intervention site compared to a control site or baseline data obtained at the same site Outcome: Quantified entomological and/or epidemiological and sociological indicators	Five studies: lethal ovitrap‐based interventions to control *Aedes* populations Two studies: host‐seeking female trap‐based interventions to control *Aedes* populations	Percentage of containers positive for larvae and/or pupae Total pupae/house Number of *Aedes* females collected inside houses using aspirators. Mean number of containers positive for *Ae. aegypti* larvae and/or pupae … (see Table S2 for more details)	Discussed but not reported
Mahmud 2023	The application of environmental management methods in combating dengue: a systematic review	Until January 2021	A total of 16 studies Among these, there were 7 RCTs, which comprised 3 cRCTs and 4 individual RCTs. The remaining 9 articles were observational studies, including 8 cross‐sectional studies and 1 case–control study	Types of studies: Cross‐sectional studies, case–control studies, RCTs, cRCTs Intervention(s) of interest: Environmental modification: Long‐lasting physical transformations to reduce vector larval habitat Environmental manipulation: … (see Table S2 for more details)	A total of eight studies that utilised non‐environmental management methods for dengue control. These methods included chemical control, biological control, or personal protection strategies	CI, HI, BI, PPI. Dengue incidence and dengue cases	RCTs were evaluated using the Cochrane Risk of Bias tool (no summary reported). Based on the supplementary provided: 3 out 7 studies had unclear risk of bias in ‘Blinding of participants and personnel (performance bias)’ domain. All other domains for all 7 studies were deemed as low risk
Maoz 2017	Community effectiveness of pyriproxyfen as a dengue vector control method: A systematic review	Search until 1 August 2016 with no starting time limit	3 RCTs and 1 cRCT out of 17 studies 17 studies in 10 countries with (3091 intervention household, 115 intervention tanks) and (2899 control household, 53 control tanks)	Studies providing original research dealing with the community effectiveness of pyriproxyfen‐alone or in combination with other chemical vector control products As for study types, included were any cRCTs or RCT; non‐RCT only if they were relevant to the research question and using a control 3. Any study that applied pyriproxyfen in the field‐defined as any community or environment where dengue vectors naturally occur—was considered community effectiveness and included in the analyses	Evaluation of pyriproxyfen‐treated devices on *Ae. aegypti* populations within a dengue‐endemic village Characterise the resistance status of *Ae. aegypti* larvae from Martinique to conventional and alternative insecticides and assess their efficacy and residual activity in simulated and field conditions Evaluate the efficacy of an experimental fumigant formulation against *Ae. aegypti* in the field, and the residents' acceptance of it together with its role in community participation for indoor control activities To determine whether covering the lids of domestic water storage containers with OlysetW Net would help controlling *Ae. aegypti* To test the effectiveness of applying an IGR in flower vases and ant traps inside and around houses. To examine the abundance of immature *Ae. aegypti* before and after the intervention, and also compare the change in abundance between the trial and control areas	Adult EI (90%) adult mortality (100%) EI%; RD = residual density of *Ae. aegypti* pre and post Only BGsentinel trap count was significant: adult density parity % CI; HI pupae/container anti Dengue IgM and IgG	No assessment conducted
Montenegro‐Quiñonez 2023	Interventions against *Aedes*/dengue at the household level: a systematic review and meta‐analysis	From May to July 2021, with an update done in February 2023	RCT: 29 studies Intervention with control: 22 studies The systematic review and meta‐analysis included a total of 61 studies conducted across 28 countries. These studies focused on various interventions at the household level against *Aedes* mosquitoes and dengue, utilising households as the primary unit of allocation. No specific no of control households	The study focuses on *Aedes* mosquitoes or dengue as a disease Follow a clear methodology, including studies containing at least one control element (e.g., RCT, cRCT, and before–after studies) Focus either on structural housing aspects or on interventions in or around the house Consider the house as the allocation unit, allowing the household members to apply the intervention(s) autonomously … (see Table S2 for more details)	The systematic review categorised the included studies based on the type of vector interventions at the household level. Here's how the interventions were divided, along with the number of studies for each type: Interventions against immature mosquito stages only: 21 studies Interventions against adult mosquitoes only: 29 studies Combined interventions against both immature and adult mosquito stages: 11 studies	Reporting by themes using indices such as CI, BI, HI and PPI	Percentage of maximum score: The studies were scored as a percentage of the maximum possible score according to the CONSORT checklist. For RCTs all 25 items of the checklist were applied, whereas for non‐RCTs, the checklist was reduced to 19 items, excluding those specifically dealing with randomisation General findings: Nearly two‐thirds of the studies scored above 65% on the CONSORT quality analysis, indicating a relatively high quality of data … (see Table S2 for more details)
Tortosa‐La Osa 2022	Effectiveness of environmental interventions to reduce entomological indices of dengue, Zika and chikungunya vector	Published between 2010 and 2020	Six studies were cRCT among the 7 studies included: Twenty clusters (neighbourhoods with approximately 200 houses) with 2 different transmission patterns were selected: 10 with high endemism and 10 with low endemism. 4 high and 4 low were randomly selected and randomly assigned by blocks (2 high and 2 low) to the control and intervention group … (see Table S2 for more details)	The inclusion criteria were: – Type of study: experimental studies (randomised or quasi‐experimental trials), published between 2010 and 2020 in Spanish, English or Portuguese – Interventions: environmental management interventions (modification, manipulation or structural changes in housing and behaviour) for dengue, Zika and chikungunya control– Outcome measures: indicators measuring the burden of disease, such as prevalence, incidence, mortality and entomological indicators … (see Table S2 for more details)	External cleaning campaigns – Composting and garbage separation – Selection of household representatives as volunteers and focus group discussions – Awareness in schools – Talk about solid waste management and composting – Coordination with authorities for garbage collection 2 Meetings with community groups, selection of women from self‐help groups to mobilise the community, focus group discussions and interviews with key informants – Distribution of educational and communication material – Meshes in the water tanks – Disposal and recycling of containers – Cleaning‐up campaigns 3 Tyre disposal – Holes drilling in trash cans – Active education in the community 4 … (see Table S2 for more details)	PPP reduction average, PPI, BI, CI, pupae per hectare index, HI	‘Checklist for randomised controlled trials’ recommended by the Cochrane collaboration and ‘checklist for quasi‐experimental studies’ published by Joanna Briggs in general, a moderate‐to‐low risk of bias, being the item related to the blinding of participants and personnel where the greatest risk was detected, which could, in certain way, be related to the characteristics of the interventions carried out. … (see Table S2 for more details)

Abbreviations: BI, Breteau index; C, control; CI, container index; cRCT, cluster randomised controlled trials; EI%, percent adult emergence inhibition; HI, house index; KAP, knowledge, attitude and practises; PI, pupae index; PPI, pupae per person index; PPP, pupae per 100 persons index; T, treatment.

**FIGURE 2:**
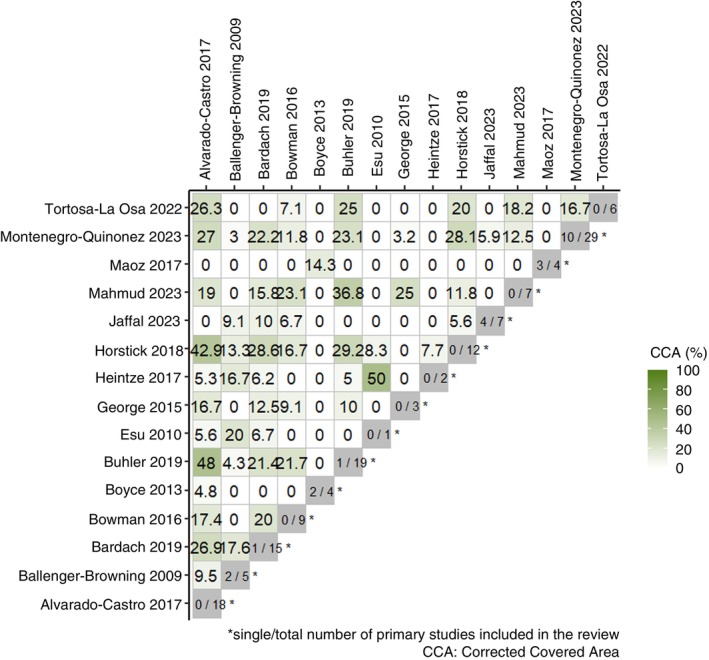
Heatmap for the Corrected Covered Area (CCA) % of all paired comparisons.

**FIGURE 3:**
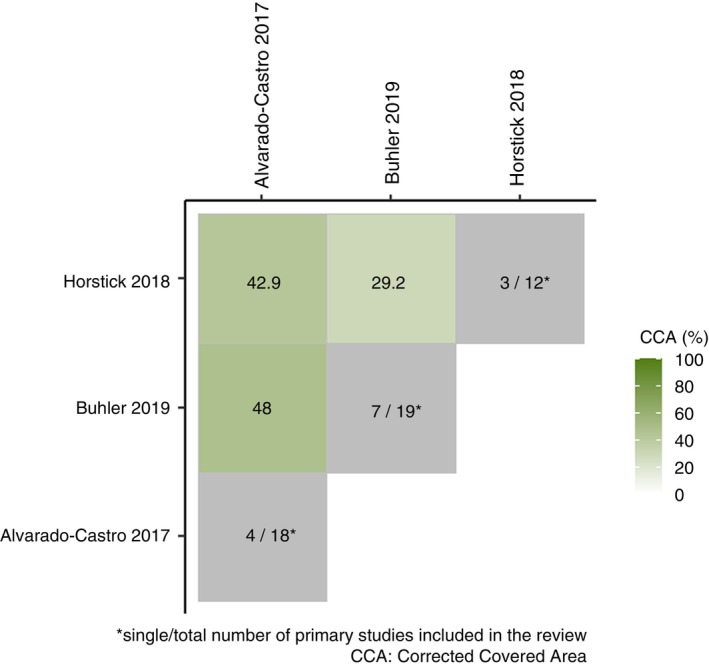
Heatmap of the CCA % among Alvarado‐Castro et al., Horstick and Runge‐Ranzinger and Buhler et al.

Overall CCA was 10.2%. Twenty‐eight of the pairwise comparisons of included studies have more than 15% of CCA (26/106 pairs). Figure 2 displays the heatmap for the CCA % of all paired comparisons. The pairwise comparisons of CCA of all included studies are tabulated in Table S3. Alvarado‐Castro et al. overlapped 42.9% and 48% of CCA with Horstick and Runge‐Ranzinger and Buhler et al., respectively. The CCA was 29.2% between Horstick and Runge‐Ranzinger and Buhler et al. Buhler et al. have 7 out of 19 primary studies that were not reviewed in Alvarado‐Castro et al. and Horstick and Runge‐Ranzinger. Figure 3 is a heatmap of the CCA % among the three systematic reviews. The overlap matrix of primary studies in each of the systematic reviews included is displayed in Table S4.

Alvarado‐Castro et al. and Horstick and Runge‐Ranzinger had more than 80% score indicating a good quality assessment in AMSTAR 2. However, Buhler et al. achieved 40.6% score in the quality assessment. All other reviews' assessments were tabulated in Table S5.

### 3.1 The Outcome Reported by Three Included Systematic Reviews Which Included RCTs and cRCTs Only as Primary Studies

Alvarado‐Castro et al. summarised the findings under three types of intervention: chemical control interventions, biological control interventions, and community participation and community mobilisation interventions. In their meta‐analysis, community mobilisation was concluded as consistently effective with an overall intervention effectiveness of −0.10 (95% confidence interval [CI] −0.20 to 0.00) for HI, −0.03 (−0.05 to −0.01) for CI, and −0.13 (−0.22 to −0.05) for BI. However, chemical control interventions were not significant: the overall effectiveness was −0.01 (−0.05 to 0.03) for HI, 0.01 (−0.01 to 0.02) for CI, and 0.01 (−0.03 to 0.05) for BI. Only a single study represents the biological control intervention with an effectiveness of −0.02 (−0.07 to 0.03) for HI, −0.02 (−0.04 to −0.01) for CI and −0.08 (−0.15 to −0.01) for BI.

Horstick and Runge‐Ranzinger concluded that single interventions that are well delivered have been effective in controlling vectors, but there is insufficient evidence in controlling transmission effectively. This is due to the lack of transmission measures reported as the study outcome in the design. Horstick and Runge‐Ranzinger found that indoor spraying has a weak effect in controlling vectors, but insecticide‐treated materials (i.e., curtains and screens) seem to have a sizeable effect on the vector, depending on the housing structure. The management of larval habitats with biological and chemical methods is useful, but environmental management and clean‐up campaigns can only be recommended because they are environmentally justifiable.

Buhler et al. concluded that both systematic review and meta‐analysis showed a weak effect of the environmental dengue vector control interventions (container coves, waste management and elimination of breeding places) on larval populations, with no obvious differences between the results of each method. Buhler et al. used difference‐in‐differences (DID) and difference‐of‐endlines (DOE) as the outcome measures in their meta‐analysis. The authors found that both container covers without insecticides (BI: DID −7.9, DOE −5) and waste management with direct garbage collection (BI: DID −8.83, DOE −6.2) achieved the strongest reductions for the BI, whereas the PPI results were almost opposite, with container covers with insecticides (PPI: DID −0.83, DOE 0.09) and elimination of breeding places (PPI: DID −0.95, DOE −0.83) showing the strongest effects. The summary findings of all other systematic reviews are tabulated in Table 2.

**TABLE 2:** Summary of findings/conclusions for each of the included systematic reviews.

Study ID	Findings/conclusion
Alvarado‐Castro 2017	Community mobilisation (four studies) was consistently effective, with an overall intervention effectiveness estimate of −0.10 (95% CI −0.20 to 0.00) for HI, −0.03 (95% CI −0.05 to −0.01) for container index, and −0.13 (95% CI −0.22 to −0.05) for BI. The single cRCT of biological control had effectiveness of −0.02 (95% CI −0.07 to 0.03) for HI, −0.02 (95% CI −0.04 to −0.01) for container index and −0.08 (95% CI −0.15 to −0.01) for BI. The five studies of chemical control did not show a significant impact on indices: the overall effectiveness was −0.01 (95% CI −0.05 to 0.03) for HI, 0.01 (95% CI −0.01 to 0.02) for container index, and 0.01 (95% CI −0.03 to 0.05) for BI.
Buhler 2019	Both, systematic review and meta‐analysis, showed a weak effect of the interventions on larval populations, with no obvious differences between the results of each individual method. For the meta‐analysis, both, container covers without insecticides (BI: DID −7.9, DOE −5) and waste management with direct garbage collection (BI: DID −8.83, DOE −6.2) achieved the strongest reductions for the BI, whereas for the PPI results were almost opposite, with container covers with insecticides (PPI: DID −0.83, DOE 0.09) and elimination of breeding places (PPI: DID −0.95, DOE −0.83) showing the strongest effects.
Horstick 2018 (Dengue specific conclusion)	Global control of dengue remains difficult. The reason for the failure of vector control of dengue is highlighted by the results in this systematic review. Although single interventions that are well delivered have been effective in controlling vectors, they have been ineffective in controlling transmission because of the study design since transmission measures are mostly not included. It can be summarised that indoor spraying has a weak effect; however, insecticide‐treated materials (i.e., curtains and screens) seems to have a sizeable effect on the vector, depending on the housing structure. The management of larval habitats with biological and chemical methods is useful, but environmental management and clean‐up campaigns can only be recommended because they are environmentally justifiable. However, reductions in vector indices alone are not sufficient to predict a reduction in transmission.
Ballenger‐Browning 2009	Overall, the results of this review have demonstrated that little concrete evidence exists to support the efficacy of mosquito abatement programmes on reducing the incidence of dengue fever. Weak study designs, incongruent indices and poorly reported statistics contribute to the questionable results.
Bardach 2019	We did not identify any intervention supported by a high certainty of evidence. In consistency with qualitative evidence, health education and community engagement probably reduces the entomological indices, as do the use of insecticide‐treated materials, indoor residual spraying and the management of containers. There is low certainty of evidence supporting the use of ovitraps or larvitraps, and the integrated epidemiological surveillance strategy to improve indices and reduce the incidence of dengue. The reported degree of implementation of these vector control interventions was variable and most did not extend to whole cities and were not sustained beyond 2 years.
Bowman 2016	Based on meta‐analyses, house screening significantly reduced dengue risk, OR 0.22 (95% CI 0.05–0.93, *p* = 0.04), as did combining community‐based environmental management and water container covers, OR 0.22 (95% CI 0.15–0.32, *p* < 0.0001). Indoor residual spraying (IRS) did not impact significantly on infection risk (OR 0.67; 95% CI 0.22–2.11; *p* = 0.50). Skin repellents, insecticide‐treated bed nets or traps had no effect (*p* > 0.5), but insecticide aerosols (OR 2.03; 95% CI 1.44–2.86) and mosquito coils (OR 1.44; 95% CI 1.09–1.91) were associated with higher dengue risk (*p* = 0.01). Although 23/41 studies examined the impact of insecticide‐based tools, only 9 evaluated the insecticide susceptibility status of the target vector population during the study.
Boyce 2013	Six studies were classified as effectiveness studies, and the remaining eight examined the efficacy of Bti in more controlled settings. Twelve (all eight efficacy studies and 4 of 6 effectiveness studies) reported reductions in entomological indices with an average duration of control of 2–4 weeks. The two effectiveness studies that did not report significant entomological reductions were both cluster‐randomised study designs that utilised basic interventions such as environmental management or general education on environment control practises in their respective control groups. Only one study described a reduction in entomological indices together with epidemiological data, reporting one dengue case in the treated area compared to 15 dengue cases in the untreated area during the observed study period.
Esu 2010	Thirteen studies showed reductions in immature entomological indices that were not sustained for long periods. The remainder showed space spray interventions to be ineffective at reducing adult and/or immature entomological indices. Only one study measured human disease indicators, but its outcomes could not be directly attributed to space sprays alone. Although peridomestic space spraying is commonly applied by national dengue control programmes, there are very few studies evaluating the effectiveness of this intervention. There is no clear evidence for recommending peridomestic space spraying as a single, effective control intervention. Thus, peridomestic space spraying is more likely best applied as part of an integrated vector management strategy.
George 2015	All 11 single intervention studies showed consistently that using temephos led to a reduction in entomological indices. Although 11 of the 16 combined intervention studies showed that temephos application together with other chemical vector control methods also reduced entomological indices, this was either not sustained over time or–as in the five remaining studies—failed to reduce the immature stages. The community‐effectiveness of temephos was found to be dependent on factors such as quality of delivery, water turnover rate, type of water, and environmental factors such as organic debris, temperature and exposure to sunlight. Timing of temephos deployment and its need for reapplication, along with behavioural factors such as the reluctance of its application to drinking water, and operational aspects such as cost, supplies, time and labour were further limitations identified in this review. In conclusion, when applied as a single intervention, temephos was found to be effective at suppressing entomological indices, however, the same effect has not been observed when temephos was applied in combination with other interventions. There is no evidence to suggest that temephos use is associated with reductions in dengue transmission.
Heintze 2017	Evidence that community‐based dengue control programmes alone and in combination with other control activities can enhance the effectiveness of dengue control programmes is weak.
Jaffal 2023	Among the 19 selected papers, lethal ovitraps were used in 16 studies, host‐seeking female traps in 3 studies. Furthermore, 16 studies focused on the control of Ae. *aegypti*. Our review showed great heterogeneity in the indicators used to assess trap efficacy: for example, the number of host‐seeking females, the number of gravid females, the proportion of positive containers, the viral infection rate in female mosquitoes or serological studies in residents. Regardless of the type of studied traps, the results of various studies support the efficacy of mass trapping in combination with classical integrated vector control in reducing *Aedes* density. More studies with standardised methodology, and indicators are urgently needed to provide more accurate estimates of their efficacy.
Mahmud 2023	This review also revealed that implementation of environmental management to control dengue is imperative whether combined or alone that has shown feasible, economical and effective to reduce mainly the entomological indices.
Maoz 2017	The results show that pyriproxyfen can be effective in reducing the numbers of *Aedes* spp. immatures with different methods of application when targeting their main breeding sites. However, the combination of pyriproxyfen with a second product increases efficacy and/or persistence of the intervention and may also slow down the development of insecticide resistance. Open questions concern concentration and frequency of application in the various treatments. Area‐wide ultra‐low volume treatment with pyriproxyfen currently lacks evidence and cannot be recommended. Community participation and acceptance has not consistently been successful and needs to be further assessed. While all studies measured entomological endpoints, only two studies measured the reduction in human dengue cases, with inconclusive results.
Montenegro‐Quiñonez 2023	Interventions at the household level against the immature mosquito stages (21 studies, 34%) showed positive or mixed results in entomological and epidemiological outcomes (86% and 75% respectively). Combined interventions against immature and adult stages (11 studies, 18%) performed similarly (91% and 67%) while those against the adult mosquitoes (29 studies, 48%) performed less well (79%, 22%). A meta‐analysis on seroconversion outcomes showed a non‐statistically significant reduction for interventions (log odds ratio: −0.18 [95% CI −0.51 to 0.14]).
Tortosa‐La Osa 2022	In 4 out of the 6 studies, there was a statistically significant reduction of the pupae indices related to the elimination of small containers, manipulation of large tanks and cleaning outdoor spaces. These interventions are easy to implement and involve little resources, which acquires special importance regarding areas with limited resources. Although it is assumed that a reduction of mosquitoes would lead to a reduction or the risk of transmission, a little evidence proving this has been published. It would be advisable that, in addition to entomological indicators, epidemiological, environmental and sociodemographic factors would be taken into consideration, bearing in mind that mosquito density is one of the many factors that influence the transmission of these viruses. None of the papers included used disease indicators, not allowing to demonstrate if environmental interventions contribute to reduce disease burden.

Abbreviations: BI, Breteau index; CI, confidence interval; cRCT, cluster randomised controlled trials; DID, difference‐in‐differences; DOE, difference‐of‐endlines; HI, house index; PPI, pupae per person index.

### 3.2 The Outcome Reported by 12 Included Systematic Reviews Which Included Non‐RCTs as Primary Studies

Among the 12 included systematic reviews, four reported low certainty of evidence regarding the effectiveness of various interventions, including mosquito abatement programmes, health education, community engagement, insecticide‐treated materials, indoor residual spraying, container management, ovitraps or larvitraps, integrated epidemiological surveillance, and peridomestic space spraying [5, 23, 24, 25]. The remaining nine systematic reviews presented mixed findings.

Bowman et al. reported house screening, along with the combination of community‐based environmental management and water‐container covers, significantly reduced dengue incidence (odds ratio [OR] = 0.22, *p* < 0.0001). However, interventions such as indoor residual spraying, skin repellents, insecticide‐treated bed nets or traps did not impact significantly on dengue incidence (*p* > 0.5). Interestingly, insecticide aerosols (OR 2.03; 95% CI: 1.44 to 2.86) and mosquito coils (OR 1.44; 95% CI: 1.09 to 1.91) were associated with higher dengue incidence (*p* = 0.01) [9].

Boyce et al. reported that 12 out of 14 primary studies on *Bacillus thuringiensis israelensis* (Bti) demonstrated reductions in entomological indices with an average duration of control of 2–4 weeks. The two studies that did not report significant entomological reductions were both cluster‐randomised study designs [22].

George et al. reviewed 11 single intervention studies that demonstrated the use of temephos was effective in reducing entomological indices. However, this effect was not observed when temephos was applied in combination with other interventions. The review also concluded that there is no evidence to suggest that the use of temephos is associated with reductions in dengue transmission [26].

Jaffal et al. reported that various studies support the efficacy of mass trapping when combined with classical integrated vector control in reducing *Aedes* density [27]. Mahmud et al. found that environmental management was effective in lowering entomological indices [19]. Maoz et al. reported that pyriproxyfen can be effective in reducing the numbers of immature *Aedes* spp., and that combining pyriproxyfen with a second product enhances efficacy and/or persistence of the intervention, potentially slowing the development of insecticide resistance. However, evidence is lacking for the effectiveness of area‐wide ultra‐low volume treatment with pyriproxyfen. While all included studies measured entomological endpoints, only two assessed reductions in human dengue cases, and their results were inconclusive [21].

Montenegro‐Quiñonez et al. reported that household‐level interventions targeting immature mosquito stages showed positive or mixed results in both entomological and epidemiological outcomes. Combined interventions targeting both immature and adult stages performed similarly, while those targeting only adult mosquitoes were less effective. A meta‐analysis on seroconversion outcomes showed a non‐statistically significant reduction for these interventions (log odds ratio: −0.18 [95% CI: −0.51 to 0.14]) [7].

Tortosa‐La Osa et al. reported that four out of the six studies showed a statistically significant reduction in pupae indices, associated with the elimination of small containers, manipulation of large tanks, and cleaning of outdoor spaces. However, none of the included papers used disease indicators, which prevents conclusions about whether these environmental interventions contribute to reducing the disease burden [20].

## 4 Discussion

We have identified that community mobilisation and insecticide‐treated materials are weakly effective interventions in reducing the population index of *Aedes* from two good quality reviews with RCTs and cRCTs as primary studies [10, 18]. However, the non‐overlapping of RCTs and cRCTs included in their respective reviews may affect the findings. The ‘chemical control’ review performed by Alvarado‐Castro et al. have two additional studies that Horstick and Runge‐Ranzinger did not include and vice versa; one study was not included in Alvarado‐Castro et al. If those studies were included in the review by Horstick and Runge‐Ranzinger, the insecticide‐treated materials may not be effective, a similar finding to Alvarado‐Castro et al. Furthermore, each study included in Horstick and Runge‐Ranzinger review had either mixed or negative results. Among reviews that included non‐RCTs as primary studies, findings were mixed; though some dengue vector control interventions showed a tendency towards improved efficacy based on entomological indices. Nevertheless, our conclusion was based on only three systematic reviews that included RCTs and cRCTs as primary studies, as we could not isolate findings based on RCTs alone in the other 12 reviews, which also included non‐RCTs.

The weak effectiveness of dengue vector control found by Buhler et al. on environmental methods might not be conclusive due to the unsatisfactory quality assessment [11]. Furthermore, the findings could not be validated in the other systematic review due to inconsistent grouping of overlapping studies between Alvarado‐Castro et al. and Buhler et al. Specifically, the overlapping studies were not classified in a comparable way where Alvarado‐Castro et al. grouped them under ‘chemical control’, whereas Buhler et al. included them as part of ‘environmental control’. Since these studies involved both types of interventions, they could not be assessed solely as environmental control, limiting the ability to validate the findings.

Previous meta‐reviews found that biological controls seem to achieve better entomological indices reduction than chemical control, while educational campaigns can reduce breeding habitats [28]. Nevertheless, they found that the quality of evidence was mostly low to very low. This is due to the inclusion of systematic reviews that also include non‐randomised intervention studies. Our findings may not support the same conclusion due to various reasons: (1) our study only includes systematic reviews that reviewed the highest quality of study design, that is, RCTs and cRCTs, (2) only a single cRCT on biological control was reviewed in one included systematic review, requiring more evidence to substantiate the effectiveness and (3) our study has included published systematic reviews on and after 2016 which the meta‐review by Bouzid et al. would not have included at the time of publication. However, our findings that agree were the weak effectiveness of chemical controls and the efficacy that has dependencies on local settings, existing control methods and a combination of interventions. Due to the integrated vector management concept, pooling results with similar interventions might have affected the meta‐analyses due to the heterogeneity in the intervention.

It is expected that Horstick and Runge‐Ranzinger and Buhler et al. have a higher degree of CCA % overlap with Alvarado‐Castro et al. because Alvarado‐Castro et al. have a broader inclusion criterion which includes chemical control, biological control and community mobilisation. Horstick and Runge‐Ranzinger and Buhler et al. both have specific inclusion criteria that focus on the protection of houses and environmental control, respectively. In addition to that, Horstick and Runge‐Ranzinger evaluated three other diseases vector control interventions along with dengue infection. Due to the differences in the objectives of the study, the search strategies and outcomes reported among the three systematic reviews are expected to be different. Hence, despite the high degree of overlapping primary studies, it is also expected that these reviews contain unique primary studies that do not overlap. The high degree of overlap, 48% between Buhler et al. and Alvarado‐Castro et al., and 42.9% between Horstick and Runge‐Ranzinger and Alvarado‐Castro et al., may introduce bias into our conclusions. To mitigate this, we carefully examined the findings of these systematic reviews to avoid overestimating the effectiveness of vector control interventions. The year in which a systematic review is published can influence the CCA, as newer reviews may include more recent trials. However, this factor is unlikely to affect the CCA among the three main studies in our analysis, as they were published within a close timeframe over a span of 3 years, thus minimising the likelihood of excluding the latest trials.

The AMSTAR 2 assessment of the three systematic reviews on RCTs revealed that Buhler et al. have inadequate quality in the methodology. Buhler et al. did not have independent reviewers to select and extract the articles, no risk of bias assessment was performed and did not satisfactorily discuss the heterogeneity and risk of bias that could have affected the findings of the study. This affects the conclusion made and might explain the reason for having a higher number of unique primary studies that did not overlap with other reviews. To investigate selective reporting among the three reviews, we mapped and compared the outcomes summarised for seven overlapped primary studies [29, 30, 31, 32, 33, 34, 35]. None of the reviews reported differently, and thus, it is unlikely that selective reporting is present in any of these three reviews.

The design of dengue vector control interventions is inherently complex, often involving a combination of chemical, biological and environmental strategies. Implementing these interventions within a pragmatic controlled trial setting can be both logistically challenging and financially demanding. As a result, our meta‐review, which was restricted to primary studies from RCTs and cRCTs, has a major limitation: it may have excluded high‐quality non‐randomised controlled studies. Such studies can offer valuable insights into intervention effectiveness in real‐world settings. To address this gap, a separate meta‐review that includes quasi‐experimental studies is currently underway and registered in PROSPERO (CRD42022323634).

We have identified another limitation of our review where the search strategy performed might not be comprehensive. Hence, we screened through the reference list of included reviews and found one additional study to be included. We investigated this occurrence and found that this article did not specify the ‘systematic review’ design in the title, thus excluded by the reviewers at the screening stage. Nevertheless, we were able to include two reviews at the screening stage to the full‐text review stage despite that both did not specify the study design in their title. It must be emphasised that study design should be included in the title as recommended by PRISMA checklist [14].

Our findings were dependent on two levels of analysis: at the systematic review level and primary studies level included in the systematic reviews. Due to the nature of meta‐review, narrative synthesis is drawn from the findings and conclusions of the systematic reviews only. Hence, no verification is attempted to evaluate the authors of systematic reviews if they have performed rigorous assessment and analysis on the primary studies. However, our AMSTAR 2 and CCA assessment allowed us to indirectly assume that the primary studies have been reviewed thoroughly.

One of the difficulties we identified in all meta‐analyses performed among the included studies is the attempt to correctly group the intervention when it is heterogenous itself. In addition, the comparators in all primary studies also differed but were grouped as ‘control’. Due to the local setting and local government's vector management, heterogeneity in the ‘control’ cannot be disregarded unless it is examined in detail enough to be satisfactorily grouped for meta‐analysis. Adding to this complexity is the integrated vector management recommended by the WHO [36].

## 5 Conclusion

There is insufficient evidence to recommend a method of dengue vector control management. Novel dengue vector control methods are highly encouraged for urgent trials. Until then, the current respective local governments' vector control management may still play a vital role in controlling mosquito propagation and transmission of dengue infection.

## Conflicts of Interest

The authors declare no conflicts of interest.

## Supporting information


**Table S1:** List of excluded studies after full‐text review that did not include any randomised controlled trial.
**Table S2:** The study characteristics of included systematic reviews (Expanded version of Table 1).
**Table S3:** Pairwise comparisons of Corrected Covered Area (CCA) % among the included studies.
**Table S4:** Overlap matrix of primary studies.
**Table S5:** AMSTAR 2 quality assessment of included systematic reviews.
