# Supplementary material for: The Effectiveness of Dengue Vector Control: A Meta‐Review
Source: Trop Med Int Health. 2025 Aug 21;30(10):1069–86. doi: 10.1111/tmi.70018 (PMC12501563; doi:10.1111/tmi.70018)
Supplement: Supplementary file 1 — Table S1: List of excluded studies after full‐text review that did not include any randomised controlled trial. Table S2: The study characteristics of included systematic reviews (Expanded version of Table 1). Table S3: Pairwise comparisons of Corrected Covered Area (CCA) % among the included studies. Table S4: Overlap matrix of primary studies. Table S5: AMSTAR 2 quality assessment of included systematic reviews. [file TMI-30-1069-s001.docx]

**Supplementary**

S1: List of excluded studies after full-text review that did not include any randomised controlled trial.

| **Authors** | **Title** |
| --- | --- |
| Aryaprema 2023 | A systematic review of published literature on mosquito control action thresholds across the world |
| Erlanger 2008 | Effect of dengue vector control interventions on entomological parameters in developing countries: a systematic review and meta-analysis |
| Gunn 2018 | Current strategies and successes in engaging women in vector control: a systematic review |
| Hustedt 2020 | Use of pyriproxyfen in control of *Aedes* mosquitoes: A systematic review |
| Lazaro 2015 | Community effectiveness of copepods for dengue vector control: systematic review |
| Lima 2015 | Meta-analysis of studies on chemical, physical and biological agents in the control of *Aedes aegypti* |
| Samuel 2017 | Community effectiveness of indoor spraying as a dengue vector control method: A systematic review |

S2: The study characteristics of included systematic reviews (Expanded version of Table 1).

| Study ID | Review title | Date of search | No. Studies included (No. clusters, households or countries in included studies) | Inclusion criteria | Comparison interventions | Outcomes for which data were reported that could be included in an analysis | Summary of quality of evidence in reviews (risk of bias) |
| --- | --- | --- | --- | --- | --- | --- | --- |
| Alvarado-Castro 2017 | Assessing the effects of interventions for *Aedes aegypti* control: systematic review and meta-analysis of cluster randomised controlled trials | January 2003 and June 2013 (updated the search in November 2016 to cover articles published up to the end of October 2016.) | 18 studies (246 intervention clusters, 48,131 intervention households) and 288 control clusters (69,430 control households) in 13 countries) | Studies concerned directly with the impact of chemical control, biological control or community mobilisation, alone or in combination, on dengue vector parameters; studies that were cRCTs; and studies that provided information about at least one of the three standard *Aedes aegypti* indices: HI- households with larvae or pupae as a proportion of households examined; CI- containers with larvae or pupae as a proportion of containers examined; and BI- containers with larvae or pupae as a proportion of households examined. | **Chemical control interventions** **1.** 1% temephos applied to household’s water containers 3 monthly. Community removal of “removable” water containers. VS Community removal of “removable” water containers. **2**. Window curtains with lambdacyhalothrin and pyriproxyfen chips in water. (Households took out chips; not considered further.) VS Control clusters received no interventions. **3**. Insecticide (permethrin) treated bednets (ITNs) supplied to households VS No treatment for 5 months; received ITNs after 6 months. **4**. In 3 intervention clusters used a. Lethal Ovitraps with deltamethrin (LO), b. *Bacillus thuringiensis israelensis* briquettes (Bti) and c. LO + Bti. Initial education of households about dengue and vector breeding and bi-weekly visits of research team VS Initial education of households about dengue and vector breeding and bi-weekly visits of research team.  **5**. Intervention 1: Window & door nets treated with deltamethrin and water container covers treated with deltamethrin (wrong size so not used). Govt programme treated water with 1% temephos in 3 intervention and 3 control clusters. Intervention 2: After 17 months, nets replaced as needed and “productive” containers treated with temephos or discarded. Govt programme continued as above. VS Govt programme treated water with 1% temephos in 3 intervention and 3 control clusters.  **6.** Window and door nets treated with long-lasting deltamethrin formulation. Max 5 nets/households. Insecticide supposed to last two years. Routine government vector control. VS Routine government vector control including temephos available to households and deltamethrin spraying if case of dengue detected. **7.** Immediate: Window and door nets treated with deltamethrin in all 10 clusters. After 8 m in 4 clusters: Water container covers treated with deltamethrin. Routine government vector control activities continued. VS Routine government vector control activities: temephos in water containers, health education, occasional malathion space spraying. **8**. Door and window screens treated with alpha-cypermethrin. After 14 m, productive containers also treated with spinosad every 2 m. Routine government vector control continued. VS Routine government vector control: temephos in water containers, space spraying with chloropyrifos and propoxur.  **Biological control interventions** **9**. Community mobilisation meetings and recruitment of ecohealth volunteers. Either copepods or *Bacillus thuringiensis israelensis toxin* (Bti) to household’s water containers, plus screen net covers for containers. Education about dengue vector by ecohealth volunteers VS No intervention Community participation and community mobilisation interventions  **10**.a. Education. House visits by university students, educational materials (eg calendars), group meetings with video + sociodrama (47 households). b. Chemical. ULV spraying malathion & temephos to water containers (46 households). c. Education & chemical (49 households) VS d. Control. No intervention (45 households) **11**. >Stakeholder discussions, steering committee >Community working groups, action plans >Coordination between community and services >Harmonisation with local vector control plan. Government routine vector control programme continued. VS Government routine vector control programme: House inspections, temephos to water containers, space spraying with cypermethrin or cloripyriphos, health education, fines for law infringements **12**. >Stakeholder consultation meetings >Involvement of women self-help groups >Mobilisation of schools, teachers & schoolchildren >Communities distributed locally made container covers and educational materials. Routine government control services. VS Routine government control services only. Some of the trial educational materials **13**. >Building partnerships of local stakeholders >Household solid waste management promoted by household volunteers >Promoting composting of biodegradable waste >Improvement of local gov rubbish collection VS Local government services **14**. Participatory strategy: > Organisation and management structures > entomological risk surveillance. >capacity building at local & intermediate level. >community work in vector control, led by community working groups (CWGs) who visited households, planned actions. Government routine vector control programme continued VS Government routine vector control programme: House inspections, temephos to water containers, space spraying with cypermethrin or cloripyriphos, health education, fines for law infringements **15**. >Community workshops  >Mobilising elders and school children for solid waste management >Government workers encouraged covering water containers >Educational materials VS Routine government vector control programme. **16**. An integrated intervention strategy (IIS)  >Elementary school education programme >Clean Patio Safe Container programme with community volunteer activators VS Government control programme:  >Initially temephos and space spraying with insecticide  >Midway, changed to biolarvicide (Bti) and households’ education for source reduction **17**. Campaign with community members & health institutions for removal of water containers around households (bags with containers collected). Engagement of community opinion makers, leaflets, & press conference. VS Routine removal of the containers by services **18**. Community discussions of baseline evidence on vector breeding sites & infection in children. Community groups planned actions: household visits by community brigades, school activities, & community clean-up activities and events. Government control programme continued. VS Government dengue control programme: temephos in households water containers & peridomestic space spraying. | **1**. BI, CI measured monthly for 10 m in 300 households randomly selected in both intervention and control clusters **2.** HI, CI, BI, PPI measured at baseline, 4w, 4 m & 12 m (Mexico) 9 m (Venezuela). Adult dengue IgM serology at baseline & 8 m in Venezuela in approx 650 households **3.** BI, HI, CI, PPI measured at baseline, 1 m, 5 m and 12 m. IgM dengue serology measured at 12 m **4.** HI, PHI, adult index measured at baseline and twice monthly for 4 m in 10 households for each intervention cluster (total 30 households) and 10 control households  **5**. At baseline, 6w after first intervention and 6w after second intervention measured total pupae, PPI, HI, BI and CI.  **6**. BI, HI, CI and PPI measured at baseline, 6 m & 18 m. All households in control clusters; random half of households in intervention clusters **7**. Measured at baseline, 9w after first intervention, and 4-6w after second intervention: CI, HI, BI, PPI  **8**. Measured BI, CI, HI & PPI at baseline and at 5, 12, 18 and 24 m Also measured adult mosquitoes.  **9.** HI, CI, BI & PPI measured at baseline, 2 m, 4 m and 6 m.  **10**. Baseline and 6 m. Measured BI, CI, HI and positive containers/households (C+/H). **11**. HI, BI and PPI measured at several points between baseline and end at 15 m **12**. CI, BI, HI and PPI measured at baseline, 5 m and 10 m **13**. Measured PPI, HI, CI and BI at baseline, 3 m, 9 m and 15 m **14**. BI measured monthly from government surveillance figures before and during intervention from mid-2005 to Dec 2007. **15**. HI, CI, BI, PPI measured at baseline and 6 m  **16.** HI, BI and PPI measured at baseline and 12 m **17.** BI, CI, HI, PPI & PHI measured at baseline and 5 m (1 m after intervention) **18.** HI, CI, BI, PPI & IgM dengue saliva serology measured at baseline, 12 m, and 15 m (Mexico) 17 m (Nicaragua) | Cochrane approach:  six studies in the meta-analysis as having a “low risk of bias” and four as having an “unclear risk of bias”  **Blinding of participants & personnel**: 18 low risk of bias **Blinding of outcome assessment**: 17 unclear risk of bias, 1 low risk of bias **Incomplete outcome data**: 10 unclear risk of bias, 8 low risk of bias **Selective reporting**: 3 unclear risk of bias, 15 low risk of bias **Other sources of bias**: 18 unclear risk of bias |
| Buhler 2019 | Environmental methods for dengue vector control – A systematic review and meta-analysis | The literature search was performed until 30th May 2017 | 19 for qualitative synthesis and 16 for meta-analysis.  Except one RCT, all studies were designed as cRCTs.  Geographically, most studies were conducted in South America (42%) and Central America (42%), with the remainder in South Asia (18%) and Southeast Asia (21%). The sample size varied from two to 75 study clusters. Duration of the interventions ranged from six weeks to 30 months with seven studies with a duration over one year, and the median duration was 6 months. | The eligibility criteria for the assessed literature were: (i) randomised controlled trials (RCTs) or cluster randomised controlled trials (cRCTs) with the following environmental vector control methods: (a) container covers without insecticides, (b) container covers with insecticides, (c) waste management with direct garbage collection, (d) waste management without direct garbage collection and (e) elimination of breeding places, (ii) outcome measures for pupal or larval indices, (iii) field studies conducted where dengue vectors naturally occur. | **1.** Twelve studies combined two or more vector control methods. Container covers without insecticides were implemented in eight studies, more specifically all these studies used lids or nets on water storage containers. In six studies, container covers were implemented in parallel with waste management campaigns. **2.** Four studies installed container covers with insecticides. Kroeger et al. applied lambdacyhalothrin to curtains in Mexico, whereas the other study by Kroeger et al. in Venezuela used firstly deltamethrin and after five months additionally lambdacyhalothrin was applied. Two studies treated water with Temephos and pyriproxyfen chips. **3.** The waste management category was divided into two subcategories, with direct garbage collection (eight studies) and no garbage collection (five studies). **4.** Elimination of breeding places was used in five studies. Applied interventions included emptying buckets, periodic inspection of houses and elimination or protection of disposable items and discarded or unused water holding containers. **5.** Seven studies also implemented non-environmental methods as insecticide-treated curtains for windows and doors, copepods, *Bacillus thuringiensis israelensis*, fish, dragonfly nymphs, mosquito traps, portable vacuum aspirators, cotton net sweepers and container treatment with insecticides. **6.** The control groups of four studies were untreated and no dengue vector control interventions were conducted. All other studies used routine programmes as control. **7.** Most of them were already existing governmental dengue vector control programmes as entomological surveillance, health education, source reduction through periodic house inspections and application of larvicides and adulticides. | A total of 17 studies reported BI, 15 studies reported HI, 13 studies reported CI, 15 studies reported PPI and five studies reported the number of positive containers. Only the study by Overgaard et al. reported the number of mosquitoes per hour inside schools collected with a Prokopack aspirator. Three studies reported measures for human transmission as the number of pupil absence periods at school, cluster specific rates of dengue virus infection in paired saliva samples from children and the number of reported dengue cases and IgM serology. | CONSORT on reporting structure. No overall conclusion |
| Horstick 2018 | Protection of the house against Chagas disease, dengue, leishmaniasis, and lymphatic filariasis: a systematic review | 1980 to 2017 | 12 out of 32 studies were dengue studies:  **Number of studies**: 12 **Number of households assessed**: Between 187 and 18,838 **Duration of follow-up**: Between 4 weeks and 24 months | Types of diseases included vector-borne NTDs such as Chagas disease, dengue, leishmaniasis (both cutaneous and visceral), and lymphatic filariasis. Furthermore, vector control operations are applied in and around households. Types of studies were RCTs and cRCTs. The types of interventions included any intervention that aimed to reduce disease incidence through vector or reservoir control in and around a house or dwelling. Insecticides for residual sprayings (intradomiciliary or peridomestic and insecticide-treated materials such as nets and curtains), and the control of larval breeding with environmental, biological, or chemical methods in and around the household, were included. The types of outcome measures encompassed disease incidence, other measurements of human disease, and entomological indicators. | **1.** the combination of indoor malathion ultra-lowvolume spraying with an educational campaign  **2**. insecticide-treated curtains  **3**. insecticide-treated curtains in open housing structures.  **4**. insecticide treated-screens in combination with Spinosad (a type of insecticide).  **5**. permanently mounted, insecticide treated screens fitted to the doors and windows of residential houses.  **6**. insecticide-treated curtains (windows or doors) alone or in combination with insecticide-treated water container covers  **7**. combination of *Mesocyclops aspericornis*, *Bacillus thuringiensis* var *israelensis* toxins, screen net covers for water jars, mosquito traps, and portable vacuum aspirators.  **8**. environmental management strategies (emptying, brushing, or scrubbing the interior walls, or covering of receptacles hosting mosquito eggs or larvae, clean-up campaigns and the use of larvivorous fish, and community mobilisation).  **9**. waste management as a single intervention  **10**. a combination of water-container covers and clean-up campaigns, including community mobilisation, on the PPI (x2)  **11**. Biogents-sentinel traps. | larvae-positive containers in households, PPI, HI, CI and BI, IgM seroprevalence (measured in a house-to-house serosurvey in Trujillo)  adult density | Cochrane Collaboration’s tool for assessing risk of bias and were scored.  **Specifically for dengue control**: 3 low quality was excluded.  11 studies were deemed medium quality and 1 study as high quality. |
| Ballenger-Browning 2009 | Multi-modal *Aedes aegypti* mosquito reduction interventions and dengue fever prevention | Searches in PubMed, Science Direct, Google Scholar, and Cab Direct databases were conducted from July 2007 to January 2008. | 5 relevant studies (RCT/cRCT).  RCT; n=187 houses (45 controls) RCT; T1:n=64 peri-domestic drums 36 with routine water use, 18 with no water use (10 controls) T2:n=28 tires (10 controls) T3:n=68 flower vases at cemetery (23 controls) CRT; T1:n=18 clusters of houses, 1095 houses (nine clusters controls) T2:n=18 clusters of houses, 1122 houses (nine clusters controls) CRT; n=12 four-block communities (six controls) CRT; *n*=30 houses at two neighbourhoods in two municipalities (one neighbourhood per municipality control) | Articles were included if an intervention was implemented and quantitative pre/post-data on larval or adult indices was reported. Studies measuring mosquitoes other than A. *aegypti* or A. *albopictus* were excluded. | T3: educational campaign only, C: no treatment; T1: malathion ULV spraying,T2: malathion spraying and educational campaign,C: no treatment . T: door-to-door education campaign;C: no treatment . T1: insecticide-treated net curtains and pyriproxyfen in water containers,C1: untreated neighbourhood;T2: PermaNet insecticide-treated curtains and covered water containers,C2: untreated neighbourhood. T1: ovitrap with deltamethrin insecticide,C1: untreated neighbourhood,T2: same as T1,C2: same as C1. Biological: *Mesocyclops longisetus* copepods:T1: peri-domestic drums 36 with routine water use, 18 with no water use. T2: tires T3: flower vases at cemetery. | BI and KAP CI BI, HI, PPI and serological testing BI and HI CI, Adult Density Index | Not done |
| Bardach 2019 | Interventions for the control of *Aedes aegypti* in Latin America and the Caribbean: systematic review and meta-analysis | January 2000 to September 2016 | A total number of nine cluster randomised controlled trials (RCTs), of 15 relevant trials, could be meta-analysed. (out of 75 included studies)  1. 32 clusters consisting of 500 houses and 2000 inhabitants in Guantanamo 2. Costarena city, two neighborhoods 3. City of Manaus. 12 clusters. 1487 houses 4. Guantanamo.12 clusters (500 homes approximately) 5. La Lisa, Havana. 16 intervention clusters (389 houses) 6. Guantanamo. 12 clusters of 500 houses each 7. Poptun. 10 experimental clusters and 10 control clusters. 2357 houses 8. Colima. 187 houses grouped into 4 blocks 9. Merida. East, South sub-areas 10. City of Renacimiento, Acapulco. 11. Acapulco. 20 clusters. 12. Veracruz and Trujillo 13. 60 clusters in Nicaragua and 90 in Mexico. In Mexico, the population is from Costa Grande, Acapulco and Costa Chica. In Nicaragua, the population is from Managua 14. Venezuela, Mexico, Peru, Kenya, Thailand, Myanmar, Vietnam and Philippines 15. Not reported but assume to be Toledo 2007. | Experimental, quasi-experimental and observational studies, economic assessments and qualitative studies related to control interventions on diseases transmitted by the *Aedes aegypti* mosquito, such as dengue, zika, chikungunya and yellow fever were considered. Studies conducted since 1995, assessing the control strategies. Any epidemiological design, from LAC countries, reporting about the effectiveness or degree of implementation of vector control interventions of any kind. The outcomes under consideration were: incidence and morbimortality of *Aedes aegypti*-related diseases, larval indices for monitoring the effect of control strategies including BI, HI and PPI, and degree of implementation or coverage levels by jurisdiction. These density indices are globally the most used in surveillance. We also assessed other vectoral indices such as recipient productivity, adult population estimation and ovitrap positivity rate. | 1. Biogents Sentineltraps (BGS) 2. Adulticides and larvicides in the ﬁeld 3. Community engagement 4. Insecticide-treated bed nets and curtains 5. Entomological surveillance as part of a Control Program 6. Insecticide-treated bednets and curtains 7. Entomological surveillance as part of a Control Program 8. Use of insecticides (larvae and adults) in the field 9. Curtains and tulle screens soaked in insecticide 10. Curtains and tulle screens soaked in insecticide 11. Community engagement 12. Insecticide-treated bednets and curtains 13. Community Engagement – Reduction in reservoirs 14. Insecticides for indoor use, reduction in reservoirs, health team training | *Aedes aegypti* population density by questionnaire to inhabitants. Serological survey Mortality of adult mosquitoes BI, HI engagement, knowledge, perception and behaviour Number of positive containers per house Reservoir Index Infestation index Overdispersion index Specific dengue infection rate (saliva samples) in children aged 3– 9 years. CI, PPI | Cochrane Handbook: RCTs are of moderate or low methodological quality in most domains explored, except for the domain related to blinding of evaluators, where the risk of bias was generally low. |
| Bowman 2016 | Is Dengue Vector Control Deficient in Effectiveness or Evidence?: Systematic Review and Meta-analysis | Publication dates: 1986 to 2014 Median year of publication: 2009 | 41 studies  Study designs: RCTs: 9 (including 7 cRCTs and 2 RCTs) Non-randomized studies: 32 Duration of studies: 5 months to 10 years Less than 1 year: 16 studies 1 to 3 years: 12 studies 8 or more years: 7 studies | **Study Design**: Studies of any design published since 1980 were included. **Target Vectors**: Studies had to evaluate methods targeting *Aedes aegypti* or Ae. *albopictus*. **Duration**: The studies needed to have a duration of at least 3 months.  **Outcomes**: Any study with empirical data reporting dengue incident data and/or entomological indices monitored longitudinally for the duration of the intervention. Dengue cases reported either by the study or obtained form external institutions (e.g., hospital records) | 1. Community-based advocacy, awareness CWGs, clean up, container covers, education (on use of larvicide), house inspections, water pipe repair  2. Water Tank Covers  3. CWGs for covering of water sources; not protecting artificial containers; not removing abate from drinking water  4. Community-based environmental management and water covers  5. Insecticide-treated curtains (x3)  6. BGS traps  7. Community participation to support existing campaigns | CI, HI, BI, PPI. Dengue incidence and dengue cases. | Cochrane risk of bias tool for RCTs: Only 9 out of 41 studies were RCTs, indicating a limited representation of high-quality evidence. Most RCTs exhibited a high risk of bias, particularly due to inadequate blinding. Selective outcome reporting was low risk in 9 studies, while incomplete outcome data had a low risk in 7 studies, with one study at medium risk and another at high risk. Allocation concealment was low risk in one study, unclear in four, and high in four. The generation of allocation sequence risk was low in four studies, unclear in four, and high in one. |
| Boyce 2013 | *Bacillus thuringiensis israelensis* (Bti) for the control of dengue vectors: systematic literature review | The literature search and analysis was developed and carried out through March 2012 | 4 out of 14 with 3 cRCTs and 1 RCT.  14 studies (368 interventional households, 4 interventional groups, 566 interventional containers, 31 intervention construction sites) and (92 control houses, 5 residential control areas, 227 control containers) in 8 countries. | **1.** research with an experimental design producing primary quantitative data,  **2.** research conducted in the field, defined as any community or environment where dengue vectors naturally occur,  **3**. the use of Bti as a single agent to control dengue vectors,  **4.** clear information on Bti formulation and dosing,  5. outcome measures reported as immature indices (i.e. Stegomyia indices, oviposition indices and/or presence/absence of immature stages of *Aedes*) and  **6.** a minimum follow-up period of at least 20 days. | **Specific objective of the 4 studies**:  **1**.Characterise the resistance status of A. *aegypti* larvae from Martinique to conventional and alternative insecticides and to assess the efficacy and residual activity under simulated and ﬁeld conditions  **2.**Compare the efﬁcacy of Abate (temephos) and Vectobac G against *Aedes* *albopictus* in bromeliads in the ﬁeld.  **3.** Evaluate two control methods for A. *aegypti* that can be used by the community: lethal ovitraps and Bti briquettes.  **4.** Determine the inﬂuence of climate and of environmental vector control with or without insecticide on *Aedes aegypti* larval indices and pupal density | Larva density at 2 weeks, 4 weeks, 6 weeks post intervention vs control group (without Bti intervention).  Length of time until tank reinfested for treated vs untreated tank.  Length of time until tank reinfested for tank treated with different concentration of Bti. Hi, CI, OI measured at first, second and third month. HI, BI, CI measured and compare between interventional and control groups. | CONSORT 2010 checklist: no risk of bias assessed. |
| Esu 2010 | Effectiveness of peridomestic space spraying with insecticide on dengue transmission; systematic review | Search conducted July 2008 | 1 cRCT study out of 15 studies included, from 10 countries. (1949 intervention household). (45 control household). 5 studies without control group. 6 studies did not clearly state the number of interventions hosehold or cluster or containers involved.  1 cRCT: Randomly selected houses drawn from approximately 7000 houses were assigned to 1 of 4 possible study arms: education alone, *n*=47 houses; ducation+ULV, *n*=49; ULV alone, *n*=46; no intervention, *n*=45) ULV: Spraying 95% malathion with truck-mounted generator on 2 occasions with a 2-month interval. Education: House-to-house visits and group meetings to educate about dengue and how to prevent dengue vector breeding. | Peer-reviewed publications that presented original data from field studies evaluating the effect of peridomestic space spraying on reducing wild *Aedes* vector populations and interrupting dengue transmission. RCTs, cRCTs, quasi-randomized controlled trials, controlled before and after studies and interrupted time series studies. The threshold of study selection was lowered to include controlled before and after studies, interrupted time series studies, post-intervention studies with a control and before and after studies without a control because of concern regarding the availability of an adequate number of studies with epidemiologically robust designs. | Cluster randomized trial:  Evaluation of an educational campaign for the elimination of *Ae. aegypti* breeding sites based on community participation, compared with ULV insecticide spraying (alone or in combination with community participation). | BI | No assessment conducted. |
| George 2015 | Community-Effectiveness of Temephos for Dengue Vector Control: A Systematic Literature Review | Searches up to 15 June 2013 | 3 relevant studies (RCT/cRCT):  Cluster RCT: 20 clusters (1835 houses)18 months  Randomized control trial Experimental area: 17,994 houses (665 blocks); Control area: 37,955 houses (1775 blocks)10 months.  Cluster RCT 16 intervention clusters (400 houses total) and 16 control clusters (400 houses total) 1 year. | **1.** Studies or programmes conducted with aim to prevent /control dengue; **2.** Studies with quantitative outcomes such as BI, CI, HI, larval mortality indicated by pupal skins, average number of positive containers per house, pupal index, indoor resting density, ovitrap index or dengue incidence;  **3.** Community effectiveness studies; **4.** Peer reviewed studies with the study designs such as RCT, cRCT, Non-RCT, Before and After studies, Studies with an Intervention and a Control area (Intervention studies); **5.** Studies where temephos was used as a single intervention or in combination with other interventions. | 1. To test the efficacy, cost and feasibility of a combined approach of insecticide treated materials alone and in combination with targeted breeding site interventions.  2. To evaluate the efficacy of temephos for the control of the Ae.*aegypti* larvae.  3. To assess the effectiveness of an integrated community based environmental management strategy to control Ae. *aegypti*, compared with a routine strategy. | HI, CI and BI were used to measure the outcomes. PPI and adult mosquito density were also measured.  Total production of *Aedes* pupae.  Pupae per person | No assessment conducted. |
| Heintze 2017 | What do community-based dengue control programmes achieve? A systematic review of published evaluations | up to March 2005 | Number of Studies: 11 (2 RCTs)  Countries: The studies were conducted in various locations, with five studies in the Americas and six in Asia. Households: The number of households targeted ranged from 5913 houses in one study to 100,000 individuals in another. Clusters: Specific details on the number of clusters were not uniformly reported across all studies, but some studies involved multiple communes or villages. | **Original Data**: Publications must present original data from trials evaluating the effect of community-based dengue control interventions. **Community-Based** **Definition**: The intervention must target the community, meaning at least one component should involve community participation (e.g., educational meetings, involvement of local leaders). **Aim of the Intervention**: The primary aim should be to reduce the incidence of dengue disease or infestation of the community with *Aedes* mosquitoes, as measured by any entomological index. **Study Design Quality**: The study design must be of acceptable quality regarding the assessment of the outcome of an intervention. **Acceptable designs included**: RCT Controlled Clinical Trials, Controlled Before and After Trials, Interrupted Time Series **Comparison Requirement**: Studies must provide some kind of comparison between the intervention setting and a non-intervention setting. | 1. Exclusively community-based dengue control.  2. Community-based dengue control in combination with chemical larvicides. | HI, BI, CI. | The quality of the studies was scored on a scale from 0 to 8, with 8 indicating the highest quality.  2 RCTs were rated as 7. |
| Jaffal 2023 | Current evidences of the efficacy of mosquito mass-trapping interventions to reduce *Aedes aegypti* and *Aedes* *albopictus* populations and *Aedes*-borne virus transmission | searches were conducted on 25 February 2021 | 7 RCT out of 19 studies.  **1.** Intervention area: group of 30 houses with LO Control: A group of 30 houses without LOs Both areas are located in the same neighbourhood: no buffer zone between intervention and control houses.  **2**. Intervention area: group of 50 houses with LO Control: group of 50 houses without LOs Both areas are located in the same village but separated by 250 m: buffer zone between intervention and control house.  **3**. Intervention area: Arcadio (150 m radius; 179 houses) Control: Santa Ana (150m radius, 164 houses)  **4**. CDC-AGO campaign was associated with an integrated vector control program. Intervention area: 23.1 Km2 (61,511 inhabitants and 25,363 houses) No control area.  **5**. Intervention area: 3 intervention clusters Control: 3 non-intervention clusters 104 to 150 houses per cluster; with a mean of 129 houses and a total of 775 houses.  **6**. 6 intervention and 6 non-intervention clusters (103– 151 households per cluster.  **7**. 3 intervention houses and 3 non-intervention houses. | Peer-reviewed articles published before February, 25 2021.  **Target population**: *Aedes* *albopictus* or *Aedes aegypti*. **Intervention**: Use of lethal ovitraps or host-seeking female traps to control mosquito populations. **Comparator**: Intervention site compared to a control site or baseline data obtained at the  same site. **Outcome**: Quantified entomological and/or epidemiological and sociological indicators | Five studies: lethal ovitrap-based interventions to control *Aedes* populations    Two studies: host-seeking female trap-based interventions to control *Aedes* populations. | Percentage of containers positive for larvae and/or pupae  Total pupae/house.  Number of *Aedes* females collected inside houses using aspirators.  Mean number of containers positive for Ae.*aegypti* larvae and/or pupae  Number of Ae.*aegypti* females collected insidehouses using aspirators.  Number of Ae.*aegypti* females captured per week usingBG-Sentinel or Stationary AGO (SAGO)traps. | Discussed but not reported. |
| Mahmud 2023 | The application of environmental management methods in combating dengue: a systematic review | Until January 2021 | A total of 16 studies.  Among these, there were 7 RCTs, which comprised 3 cRCTs and 4 individual RCTs. The remaining 9 articles were observational studies, including 8 cross-sectional studies and 1 case-control study | **Types of Studies**: Cross-sectional studies, Case-control studies, RCTs, cRCTs  **Intervention(s) of Interest**: Environmental modification: Long-lasting physical transformations to reduce vector larval habitat. Environmental manipulation: Temporary changes to vector habitats involving the management of essential containers. Human behavior: Changes in human behavior to reduce human–vector contact.  **Comparator**: Any other types of interventions without environmental management for dengue control, such as chemical control, biological control, or personal protection.  **Outcomes**: **Primary outcome**: Cases of confirmed dengue as defined by WHO guidelines. **Secondary outcomes** included various entomological indices such as HI, BI, CI, and PI. | A total of 8 studies that utilized non-environmental management methods for dengue control. These methods included chemical control, biological control, or personal protection strategies | CI, HI, BI, PPI. Dengue incidence and dengue cases. | RCTs were evaluated using the Cochrane Risk of Bias tool.  (No summary reported). Based on the supplementary provided: 3 out 7 studies had unclear risk of bias in “Blinding of participants and personnel (Performance bias)” domain. All other domains for all 7 studies were deemed as low risk. |
| Maoz 2017 | Community effectiveness of pyriproxyfen as a dengue vector control method: A systematic review | search until 01 August 2016 with no starting time limit | 3 RCTs and 1 cRCT out of 17 studies  17 studies in 10 countries with (3091 intervention household, 115 intervention tanks) and (2899 control household, 53 control tanks). | **1.** Studies providing original research dealing with the community effectiveness of pyriproxyfen-alone or in combination with other chemical vector control products.  **2.** As for study types, included were any cRCTs or RCT; non-RCT only if they were relevant to the research question and using a control. **3.** Any study that applied pyriproxyfen in the field-defined as any community or environment where dengue vectors naturally occur-was considered community effectiveness and included in the analyses. | **1**. Evaluation of pyriproxyfen-treated devices on Ae. *aegypti* populations within a dengue-endemic village.  **2**. Characterise the resistance status of A. *aegypti* larvae from Martinique to conventional and alternative insecticides and assess their efficacy and residual activity in simulated and field conditions.  **3**. Evaluate the efficacy of an experimental fumigant formulation against Ae. *aegypti* in the field, and the residents’ acceptance of it together with its role in community participation for indoor control activities.  **4**. To determine whether covering the lids of domestic water storage containers with OlysetW Net would help controlling Ae. *Aegypti* To test the effectiveness of applying an IGR in flower vases and ant traps inside and around houses. To examine the abundance of immature Ae. *aegypti* before and after the intervention, and also compare the change in abundance between the trial and control areas | adult EI (90%) adult mortality (100%)  EI %; RD = residual density of Ae. *aegypti* pre and post  only BGsentinel trap count was significant: adult density parity %  CI; HI pupae/container anti Dengue IgM & IgG | No assessment conducted. |
| Montenegro-Quiñonez 2023 | Interventions against *Aedes*/dengue at the household level: a systematic review and meta-analysis | from May to July 2021, with an update done in February 2023 | RCT: 29 studies.  Intervention with control: 22 studies  The systematic review and meta-analysis included a total of 61 studies conducted across 28 countries. These studies focused on various interventions at the household level against *Aedes* mosquitoes and dengue, utilizing households as the primary unit of allocation. No specific no of control households | The study focuses on *Aedes* mosquitoes or dengue as a disease.  Follow a clear methodology, including studies containing at least one control element (e.g., RCT, cRCT, and before-after studies).  Focus either on structural housing aspects or on interventions in or around the house.  Consider the house as the allocation unit, allowing the household members to apply the intervention(s) autonomously.  Consider interventions carried out in field situations and focused on community effectiveness. | The systematic review categorised the included studies based on the type of vector interventions at the household level. Here’s how the interventions were divided, along with the number of studies for each type:  Interventions against immature mosquito stages only: 21 studies.  Interventions against adult mosquitoes only: 29 studies. Combined interventions against both immature and adult mosquito stages: 11 studies. | Reporting by themes using indices such as CI, BI, HI and PPI. | Percentage of Maximum Score: The studies were scored as a percentage of the maximum possible score according to the CONSORT checklist. For RCTs all 25 items of the checklist were applied, whereas for non-RCTs, the checklist was reduced to 19 items, excluding those specifically dealing with randomization. General Findings: Nearly two-thirds of the studies scored above 65% on the CONSORT quality analysis, indicating a relatively high quality of data and results. Studies used in the meta-analysis generally had a higher median quality score, with all but one scoring above 70%. Risk of Bias: The review notes that studies with higher quality scores provided more reliable data and results. Lower scores generally reflected earlier or simpler studies but did not impede content analysis. Publication bias is mentioned as a limitation since negative results are less likely to be reported. However, a broad search strategy and manual searches were employed to mitigate this bias. |
| Tortosa-La Osa 2022 | Effectiveness of environmental interventions to reduce entomological indices of dengue, Zika, and chikungunya vector | published between 2010 and 2020 | 6 studies were cRCT among the 7 studies included: **1**. Twenty clusters (neighbourhoods with approximately 200 houses) with 2 different transmission patterns were selected: 10 with high endemism and 10 with low endemism. 4 high and 4 low were randomly selected and randomly assigned by blocks (2 high and 2 low) to the control and intervention group.  **2.** Twenty clusters (with approximately 100 houses with public areas) were randomly selected and matched according to ecological and entomological indices and sociological parameters collected during the baseline study.  **3**. 1442 households in Monmouth and 1251 in Mercer were selected to receive the intervention. (Quasi) **4**. Twenty clusters (100 houses each) were randomly selected and grouped into pairs according to geographical and ecological parameters, types of houses and economic, cultural and social parameters and of each pair. 10 were randomly assigned to the control group and 10 to the intervention group.  **5.** Random selection of 20 clusters, grouping by pairs according to sociological and ecological parameters, and random assignment by pair to the control and intervention groups.  **6**. Twenty clusters (of about 100 houses) were selected and grouped in pairs based on similarity of socioeconomic indicators and baseline PPI. Ten clusters were randomly assigned to the control and intervention group.  **7**. The city was divided into clusters (areas of at least 100 houses), numbered and randomly selected 20, which were randomly assigned to control (n = 9) and intervention (n = 11). | The inclusion criteria were: – Type of study: experimental studies (randomized or quasi-experimental trials), published between 2010 and 2020 in Spanish, English, or Portuguese,  – Interventions: environmental management interventions (modification, manipulation or structural changes in housing and behaviour) for dengue, Zika and chikungunya control, – Outcome measures: indicators measuring the burden of disease, such as prevalence, incidence, mortality,  entomological indicators measuring the presence and abundance of vectors, such as eggs, larvae, pupae, or adults’ rates The exclusion criteria were: – Interventions including, in addition to environmental, chemical or biological control, not providing disaggregated results. | **1**.- External cleaning campaigns - Composting and garbage separation - Selection of household representatives as volunteers and focus group discussions - Awareness in schools  - Talk about solid waste management and composting - Coordination with authorities for garbage collection **2**.- Meetings with community groups, selection of women from self-help groups to mobilize the community, focus group discussions and interviews with key informants. - Distribution of educational and communication material. - Meshes in the water tanks - Disposal and recycling of containers - Cleaning-up campaigns  **3**.- Tyre disposal  - Holes drilling in trash cans - Active education in the community **4**. - Meetings with local health authorities, municipal authorities, and political leaders - Round table with health professionals - Elimination of small containers - Removal or handling of large water tanks **5**. - Elimination of small containers - Covering of large water containers - Cleaning-up public spaces. - Identification of key individuals, community workshops. -mobilization of children and the elderly and distribution of information and educational material - Request to the Secretary for a truck for garbage collection **6**. - Elimination of small containers - Handling of large tanks - Education in schools **7**. - Elimination of small containers - Retransmission of messages to the population. - Social mobilization (only in 1 area) | PPP reduction average, PPI, BI, CI, Pupae per Hectare Index, HI | "Checklist for randomized controlled trials" recommended by the Cochrane collaboration and "checklist for quasi-experimental studies" published by Joanna Briggs. in general, a moderate-to-low risk of bias, being the item related to the blinding of participants and personnel where the greatest risk was detected, which could, in certain way, be related to the characteristics of the interventions carried out. Concerning the quasi-experimental study, an evaluation of high risk of bias was carried out due to the uncertainty generated by the process of measuring the results |

Abbreviations: cRCT= cluster randomized controlled trials; HI= house index; CI= container index; BI= Breteau index; PPI= pupae per person index; PHI= Pupae per house index; Pupae Index= PI ;KAP= knowledge, attitude and practices; T=treatment; C=control; EI%= per cent adult emergence inhibition; PPP= Pupae per 100 Persons Index

S3: Pairwise comparisons of Corrected Covered Area (CCA) % among the included studies.

| **Reviews** | **Overlap counts** | **N** | **r** | **c** | **CCA Percentage** |
| --- | --- | --- | --- | --- | --- |
| Overall |  | 141 | 57 | 15 | 0.105263 |
| Esu 2010 vs. Heintze 2017 | 1 | 3 | 2 | 2 | 0.5 |
| Alvarado-Castro 2017 vs. Buhler 2019 | 12 | 37 | 25 | 2 | 0.48 |
| Alvarado-Castro 2017 vs. Horstick 2018 | 9 | 30 | 21 | 2 | 0.428571 |
| Buhler 2019 vs. Mahmud 2023 | 7 | 26 | 19 | 2 | 0.368421 |
| Buhler 2019 vs. Horstick 2018 | 7 | 31 | 24 | 2 | 0.291667 |
| Bardach 2019 vs. Horstick 2018 | 6 | 27 | 21 | 2 | 0.285714 |
| Horstick 2018 vs. Montenegro-QuiÃ±onez 2023 | 9 | 41 | 32 | 2 | 0.28125 |
| Alvarado-Castro 2017 vs. Montenegro-QuiÃ±onez 2023 | 10 | 47 | 37 | 2 | 0.27027 |
| Alvarado-Castro 2017 vs. Bardach 2019 | 7 | 33 | 26 | 2 | 0.269231 |
| Alvarado-Castro 2017 vs. Tortosa-La Osa 2022 | 5 | 24 | 19 | 2 | 0.263158 |
| Buhler 2019 vs. Tortosa-La Osa 2022 | 5 | 25 | 20 | 2 | 0.25 |
| George 2015 vs. Mahmud 2023 | 2 | 10 | 8 | 2 | 0.25 |
| Bowman 2016 vs. Mahmud 2023 | 3 | 16 | 13 | 2 | 0.230769 |
| Buhler 2019 vs. Montenegro-QuiÃ±onez 2023 | 9 | 48 | 39 | 2 | 0.230769 |
| Bardach 2019 vs. Montenegro-QuiÃ±onez 2023 | 8 | 44 | 36 | 2 | 0.222222 |
| Bowman 2016 vs. Buhler 2019 | 5 | 28 | 23 | 2 | 0.217391 |
| Bardach 2019 vs. Buhler 2019 | 6 | 34 | 28 | 2 | 0.214286 |
| Ballenger-Browning 2009 vs. Esu 2010 | 1 | 6 | 5 | 2 | 0.2 |
| Bardach 2019 vs. Bowman 2016 | 4 | 24 | 20 | 2 | 0.2 |
| Horstick 2018 vs. Tortosa-La Osa 2022 | 3 | 18 | 15 | 2 | 0.2 |
| Alvarado-Castro 2017 vs. Mahmud 2023 | 4 | 25 | 21 | 2 | 0.190476 |
| Mahmud 2023 vs. Tortosa-La Osa 2022 | 2 | 13 | 11 | 2 | 0.181818 |
| Ballenger-Browning 2009 vs. Bardach 2019 | 3 | 20 | 17 | 2 | 0.176471 |
| Alvarado-Castro 2017 vs. Bowman 2016 | 4 | 27 | 23 | 2 | 0.173913 |
| Alvarado-Castro 2017 vs. George 2015 | 3 | 21 | 18 | 2 | 0.166667 |
| Ballenger-Browning 2009 vs. Heintze 2017 | 1 | 7 | 6 | 2 | 0.166667 |
| Bowman 2016 vs. Horstick 2018 | 3 | 21 | 18 | 2 | 0.166667 |
| Montenegro-QuiÃ±onez 2023 vs. Tortosa-La Osa 2022 | 5 | 35 | 30 | 2 | 0.166667 |
| Bardach 2019 vs. Mahmud 2023 | 3 | 22 | 19 | 2 | 0.157895 |
| Boyce 2013 vs. Maoz 2017 | 1 | 8 | 7 | 2 | 0.142857 |
| Ballenger-Browning 2009 vs. Horstick 2018 | 2 | 17 | 15 | 2 | 0.133333 |
| Bardach 2019 vs. George 2015 | 2 | 18 | 16 | 2 | 0.125 |
| Mahmud 2023 vs. Montenegro-QuiÃ±onez 2023 | 4 | 36 | 32 | 2 | 0.125 |
| Bowman 2016 vs. Montenegro-QuiÃ±onez 2023 | 4 | 38 | 34 | 2 | 0.117647 |
| Horstick 2018 vs. Mahmud 2023 | 2 | 19 | 17 | 2 | 0.117647 |
| Bardach 2019 vs. Jaffal 2023 | 2 | 22 | 20 | 2 | 0.1 |
| Buhler 2019 vs. George 2015 | 2 | 22 | 20 | 2 | 0.1 |
| Alvarado-Castro 2017 vs. Ballenger-Browning 2009 | 2 | 23 | 21 | 2 | 0.095238 |
| Ballenger-Browning 2009 vs. Jaffal 2023 | 1 | 12 | 11 | 2 | 0.090909 |
| Bowman 2016 vs. George 2015 | 1 | 12 | 11 | 2 | 0.090909 |
| Esu 2010 vs. Horstick 2018 | 1 | 13 | 12 | 2 | 0.083333 |
| Heintze 2017 vs. Horstick 2018 | 1 | 14 | 13 | 2 | 0.076923 |
| Bowman 2016 vs. Tortosa-La Osa 2022 | 1 | 15 | 14 | 2 | 0.071429 |
| Bardach 2019 vs. Esu 2010 | 1 | 16 | 15 | 2 | 0.066667 |
| Bowman 2016 vs. Jaffal 2023 | 1 | 16 | 15 | 2 | 0.066667 |
| Bardach 2019 vs. Heintze 2017 | 1 | 17 | 16 | 2 | 0.0625 |
| Jaffal 2023 vs. Montenegro-QuiÃ±onez 2023 | 2 | 36 | 34 | 2 | 0.058824 |
| Alvarado-Castro 2017 vs. Esu 2010 | 1 | 19 | 18 | 2 | 0.055556 |
| Horstick 2018 vs. Jaffal 2023 | 1 | 19 | 18 | 2 | 0.055556 |
| Alvarado-Castro 2017 vs. Heintze 2017 | 1 | 20 | 19 | 2 | 0.052632 |
| Buhler 2019 vs. Heintze 2017 | 1 | 21 | 20 | 2 | 0.05 |
| Alvarado-Castro 2017 vs. Boyce 2013 | 1 | 22 | 21 | 2 | 0.047619 |
| Ballenger-Browning 2009 vs. Buhler 2019 | 1 | 24 | 23 | 2 | 0.043478 |
| George 2015 vs. Montenegro-QuiÃ±onez 2023 | 1 | 32 | 31 | 2 | 0.032258 |
| Ballenger-Browning 2009 vs. Montenegro-QuiÃ±onez 2023 | 1 | 34 | 33 | 2 | 0.030303 |
| Alvarado-Castro 2017 vs. Jaffal 2023 | 0 | 25 | 25 | 2 | 0 |
| Alvarado-Castro 2017 vs. Maoz 2017 | 0 | 22 | 22 | 2 | 0 |
| Ballenger-Browning 2009 vs. Bowman 2016 | 0 | 14 | 14 | 2 | 0 |
| Ballenger-Browning 2009 vs. Boyce 2013 | 0 | 9 | 9 | 2 | 0 |
| Ballenger-Browning 2009 vs. George 2015 | 0 | 8 | 8 | 2 | 0 |
| Ballenger-Browning 2009 vs. Mahmud 2023 | 0 | 12 | 12 | 2 | 0 |
| Ballenger-Browning 2009 vs. Maoz 2017 | 0 | 9 | 9 | 2 | 0 |
| Ballenger-Browning 2009 vs. Tortosa-La Osa 2022 | 0 | 11 | 11 | 2 | 0 |
| Bardach 2019 vs. Boyce 2013 | 0 | 19 | 19 | 2 | 0 |
| Bardach 2019 vs. Maoz 2017 | 0 | 19 | 19 | 2 | 0 |
| Bardach 2019 vs. Tortosa-La Osa 2022 | 0 | 21 | 21 | 2 | 0 |
| Bowman 2016 vs. Boyce 2013 | 0 | 13 | 13 | 2 | 0 |
| Bowman 2016 vs. Esu 2010 | 0 | 10 | 10 | 2 | 0 |
| Bowman 2016 vs. Heintze 2017 | 0 | 11 | 11 | 2 | 0 |
| Bowman 2016 vs. Maoz 2017 | 0 | 13 | 13 | 2 | 0 |
| Boyce 2013 vs. Buhler 2019 | 0 | 23 | 23 | 2 | 0 |
| Boyce 2013 vs. Esu 2010 | 0 | 5 | 5 | 2 | 0 |
| Boyce 2013 vs. George 2015 | 0 | 7 | 7 | 2 | 0 |
| Boyce 2013 vs. Heintze 2017 | 0 | 6 | 6 | 2 | 0 |
| Boyce 2013 vs. Horstick 2018 | 0 | 16 | 16 | 2 | 0 |
| Boyce 2013 vs. Jaffal 2023 | 0 | 11 | 11 | 2 | 0 |
| Boyce 2013 vs. Mahmud 2023 | 0 | 11 | 11 | 2 | 0 |
| Boyce 2013 vs. Montenegro-QuiÃ±onez 2023 | 0 | 33 | 33 | 2 | 0 |
| Boyce 2013 vs. Tortosa-La Osa 2022 | 0 | 10 | 10 | 2 | 0 |
| Buhler 2019 vs. Esu 2010 | 0 | 20 | 20 | 2 | 0 |
| Buhler 2019 vs. Jaffal 2023 | 0 | 26 | 26 | 2 | 0 |
| Buhler 2019 vs. Maoz 2017 | 0 | 23 | 23 | 2 | 0 |
| Esu 2010 vs. George 2015 | 0 | 4 | 4 | 2 | 0 |
| Esu 2010 vs. Jaffal 2023 | 0 | 8 | 8 | 2 | 0 |
| Esu 2010 vs. Mahmud 2023 | 0 | 8 | 8 | 2 | 0 |
| Esu 2010 vs. Maoz 2017 | 0 | 5 | 5 | 2 | 0 |
| Esu 2010 vs. Montenegro-QuiÃ±onez 2023 | 0 | 30 | 30 | 2 | 0 |
| Esu 2010 vs. Tortosa-La Osa 2022 | 0 | 7 | 7 | 2 | 0 |
| George 2015 vs. Heintze 2017 | 0 | 5 | 5 | 2 | 0 |
| George 2015 vs. Horstick 2018 | 0 | 15 | 15 | 2 | 0 |
| George 2015 vs. Jaffal 2023 | 0 | 10 | 10 | 2 | 0 |
| George 2015 vs. Maoz 2017 | 0 | 7 | 7 | 2 | 0 |
| George 2015 vs. Tortosa-La Osa 2022 | 0 | 9 | 9 | 2 | 0 |
| Heintze 2017 vs. Jaffal 2023 | 0 | 9 | 9 | 2 | 0 |
| Heintze 2017 vs. Mahmud 2023 | 0 | 9 | 9 | 2 | 0 |
| Heintze 2017 vs. Maoz 2017 | 0 | 6 | 6 | 2 | 0 |
| Heintze 2017 vs. Montenegro-QuiÃ±onez 2023 | 0 | 31 | 31 | 2 | 0 |
| Heintze 2017 vs. Tortosa-La Osa 2022 | 0 | 8 | 8 | 2 | 0 |
| Horstick 2018 vs. Maoz 2017 | 0 | 16 | 16 | 2 | 0 |
| Jaffal 2023 vs. Mahmud 2023 | 0 | 14 | 14 | 2 | 0 |
| Jaffal 2023 vs. Maoz 2017 | 0 | 11 | 11 | 2 | 0 |
| Jaffal 2023 vs. Tortosa-La Osa 2022 | 0 | 13 | 13 | 2 | 0 |
| Mahmud 2023 vs. Maoz 2017 | 0 | 11 | 11 | 2 | 0 |
| Maoz 2017 vs. Montenegro-QuiÃ±onez 2023 | 0 | 33 | 33 | 2 | 0 |
| Maoz 2017 vs. Tortosa-La Osa 2022 | 0 | 10 | 10 | 2 | 0 |

Abbreviations: N= total number of included publications (including double counting) in evidence synthesis; r= number of rows (number of index publications) ; c= number of columns (number of reviews).

S4: Overlap matrix of primary studies.

| Systematic Reviews  Primary Studies | Alvarado-Castro 2017 | Buhler 2019 | Horstick 2018 | Ballenger-Browning 2009 | Bardach 2019 | Bowman 2016 | Boyce 2013 | Esu 2010 | George 2015 | Heintze 2017 | Mahmud 2023 | Maoz 2017 | Montenegro-Quiñonez 2023 | Jaffal 2023 | Tortosa-La Osa 2022 |
| --- | --- | --- | --- | --- | --- | --- | --- | --- | --- | --- | --- | --- | --- | --- | --- |
| Abeyewickreme 2012 | 1 | 1 | 1 |  |  |  |  |  |  |  |  |  | 1 |  | 1 |
| Akhoundi 2018 |  |  |  |  |  |  |  |  |  |  |  |  |  | 1 |  |
| Andersson 2015 | 1 | 1 | 1 |  | 1 |  |  |  |  |  |  |  |  |  |  |
| Arunachalam 2012 | 1 | 1 | 1 |  |  | 1 |  |  |  |  | 1 |  | 1 |  | 1 |
| Barrera 2018 |  |  |  |  |  |  |  |  |  |  |  |  |  | 1 |  |
| Barrera 2019 |  |  |  |  |  |  |  |  |  |  |  |  |  | 1 |  |
| Basso 2015 | 1 | 1 |  |  |  |  |  |  |  |  |  |  | 1 |  | 1 |
| Basso 2017 |  |  |  |  |  |  |  |  |  |  |  |  | 1 |  | 1 |
| Bigio 2022 |  |  |  |  |  |  |  |  |  |  |  |  | 1 |  |  |
| Camargo-Donalisio 2002 | 1 |  |  |  |  |  |  |  | 1 |  |  |  |  |  |  |
| Caprara 2015 | 1 | 1 | 1 |  |  |  |  |  |  |  | 1 |  | 1 |  | 1 |
| Castro 2012 | 1 | 1 |  |  | 1 | 1 |  |  |  |  |  |  |  |  |  |
| Che-Mendoza 2015 | 1 |  | 1 |  | 1 |  |  |  |  |  |  |  | 1 |  |  |
| Che-Mendoza 2018 |  |  |  |  |  |  |  |  |  |  |  |  | 1 |  |  |
| Degener 2014 |  |  | 1 |  | 1 | 1 |  |  |  |  |  |  | 1 | 1 |  |
| Degener 2015 |  |  |  |  |  |  |  |  |  |  |  |  | 1 | 1 |  |
| Devine 2021 |  |  |  |  |  |  |  |  |  |  |  |  | 1 |  |  |
| Espinoza-Gomez 2002 | 1 |  | 1 | 1 | 1 |  |  | 1 |  | 1 |  |  |  |  |  |
| Favier 2006 |  |  |  |  |  |  | 1 |  |  |  |  |  |  |  |  |
| Forsyth 2022 |  |  |  |  |  |  |  |  |  |  |  |  | 1 |  |  |
| Gorrochotegui-Escalante 1998 |  |  |  | 1 |  |  |  |  |  |  |  |  |  |  |  |
| Harburguer 2011 |  |  |  |  |  |  |  |  |  |  |  | 1 |  |  |  |
| Hustedt 2021 |  |  |  |  |  |  |  |  |  |  |  |  | 1 |  |  |
| Kittayapong 2012 | 1 | 1 | 1 |  |  |  |  |  |  |  |  |  |  |  |  |
| Kroeger 2006 | 1 | 1 | 1 | 1 | 1 |  |  |  |  |  |  |  | 1 |  |  |
| Kusumawathie 2009 |  | 1 |  |  |  | 1 |  |  |  |  |  |  |  |  |  |
| Lenhart 2008 | 1 |  |  |  |  |  |  |  |  |  |  |  | 1 |  |  |
| Lenhart 2013 |  |  | 1 |  |  | 1 |  |  |  |  |  |  | 1 |  |  |
| Lenhart 2020 |  |  |  |  |  |  |  |  |  |  |  |  | 1 |  |  |
| Lenhart 2022 |  |  |  |  |  |  |  |  |  |  |  |  | 1 |  |  |
| Leontsini 1993 |  | 1 |  |  |  |  |  |  |  | 1 |  |  |  |  |  |
| Lloyd 1992 |  |  |  | 1 |  |  |  |  |  |  |  |  |  |  |  |
| Loroño-Pino 2013 |  |  |  |  | 1 | 1 |  |  |  |  |  |  |  |  |  |
| Manrique-Saide 2015 |  |  | 1 |  | 1 |  |  |  |  |  |  |  | 1 |  |  |
| Manrique-Saide 2021a |  |  |  |  |  |  |  |  |  |  |  |  | 1 |  |  |
| Manrique-Saide 2021b |  |  |  |  |  |  |  |  |  |  |  |  | 1 |  |  |
| Marcombe 2011 |  |  |  |  |  |  | 1 |  |  |  |  | 1 |  |  |  |
| Mitchell-Foster 2015 | 1 | 1 |  |  |  |  |  |  |  |  |  |  |  |  | 1 |
| Morrison 2022 |  |  |  |  |  |  |  |  |  |  |  |  | 1 |  |  |
| Ocampo 2009 | 1 |  |  |  |  |  | 1 |  |  |  |  |  |  |  |  |
| Overgaard 2016 |  | 1 |  |  |  |  |  |  |  |  | 1 |  |  |  |  |
| Perich 2003 |  |  |  | 1 | 1 |  |  |  |  |  |  |  |  | 1 |  |
| Ponlawat 2013 |  |  |  |  |  |  |  |  |  |  |  | 1 |  |  |  |
| Quintero 2015 | 1 | 1 | 1 |  |  |  |  |  |  |  |  |  | 1 |  |  |
| Rizzo 2012 | 1 | 1 |  |  | 1 |  |  |  | 1 |  | 1 |  | 1 |  |  |
| Sithiprasasna 2003 |  |  |  |  |  |  |  |  |  |  |  |  |  | 1 |  |
| Sulaiman 1999 |  |  |  |  |  |  | 1 |  |  |  |  |  |  |  |  |
| Tana 2012 |  | 1 |  |  |  |  |  |  |  |  |  |  |  |  |  |
| Toledo 2007 |  | 1 |  |  |  | 1 |  |  |  |  | 1 |  |  |  |  |
| Toledo 2011 |  |  |  |  | 1 |  |  |  |  |  |  |  |  |  |  |
| Toledo 2015 |  |  |  |  | 1 |  |  |  |  |  |  |  | 1 |  |  |
| Toledo 2017 |  |  |  |  | 1 |  |  |  |  |  |  |  | 1 |  |  |
| Tsunoda 2013 |  |  |  |  |  |  |  |  |  |  |  | 1 |  |  |  |
| Tun-Lin 2009 |  | 1 |  |  | 1 |  |  |  |  |  | 1 |  | 1 |  |  |
| Vanlerberghe 2009 | 1 | 1 |  |  | 1 | 1 |  |  | 1 |  | 1 |  |  |  |  |
| Vanlerberghe 2013 | 1 |  |  |  |  | 1 |  |  |  |  |  |  | 1 |  |  |
| Wai 2012 |  | 1 |  |  |  |  |  |  |  |  |  |  | 1 |  |  |

S5: AMSTAR 2 quality assessment of included systematic reviews.

| Question | Alvarado-Castro 2017 | Buhler 2019 | Horstick 2018 | Ballenger-Browning 2009 | Bardach 2019 | Bowman 2016 | Boyce 2013 | Mahmud 2023 | Maoz 2017 | Montenegro-Quiñonez 2023 | Esu 2010 | George 2015 | Heintze 2017 | Jaffal 2023 | Tortosa-La-Osa 2022 |
| --- | --- | --- | --- | --- | --- | --- | --- | --- | --- | --- | --- | --- | --- | --- | --- |
| 1 | 1 | 0 | 1 | 1 | 1 | 1.0 | 1 | 1.0 | 1 | 0 | 1 | 1 | 1.0 | 1 | 1 |
| 2 | 0 | 0 | 0 | 0 | 0 | 0 | 0 | 0.5 | 0 | 0 | 0 | 0 | 0 | 0 | 0 |
| 3 | 1 | 1 | 1 | 1 | 1 | 0 | 1 | 1.0 | 1 | 0 | 1 | 0 | 1.0 | 0 | 0 |
| 4 | 0.5 | 0.5 | 0.5 | 0 | 0 | 0.5 | 0.5 | 0.5 | 0.5 | 0.5 | 0.5 | 0.5 | 1.0 | 0.5 | 0 |
| 5 | 1 | 0 | 1 | 1 | 1 | 1.0 | 0 | 1.0 | 1 | 0 | 0 | 1 | 1.0 | 1 | 1 |
| 6 | 1 | 0 | 1 | 0 | 0 | 1.0 | 0 | 1.0 | 0 | 0 | 1 | 1 | 0 | 1 | 1 |
| 7 | 1 | 1 | 1 | 0 | 1 | 1.0 | 1 | 1.0 | 1 | 1.0 | 0 | 0 | 0 | 0 | 1 |
| 8 | 1 | 1 | 1 | 1 | 1 | 0.5 | 1 | 1.0 | 1 | 1.0 | 1 | 0 | 0.5 | 1 | 0 |
| 9 | 1 | 0 | 1 | 0 | 1 | 1.0 | 0 | NA | 0 | 0 | 0 | 0 | NA | 0 | 1 |
| 10 | 0 | 0 | 0 | 0 | 0 | 0 | 0 | 1.0 | 0 | 0 | 0 | 0 | 0.5 | 0 | 0 |
| 11 | 1 | 0 | NA | NA | 1 | 1.0 | NA | 0 | NA | 0 | NA | NA | 0 | NA | NA |
| 12 | 1 | 0 | NA | NA | 0 | 1.0 | NA | 0 | NA | 0 | NA | NA | 0 | NA | NA |
| 13 | 1 | 1 | 1 | 0 | 1 | 1.0 | 0 | 0 | 0 | 0 | 1 | 0 | 0 | 0 | 0 |
| 14 | 1 | 0 | 1 | 0 | 1 | 1.0 | 0 | 0 | 0 | 0 | 1 | 0 | 0 | 0 | 0 |
| 15 | 1 | 1 | 1 | 0 | 0 | 0 | 0 | 0 | 0 | 0 | 0 | 0 | 0 | 0 | 0 |
| 16 | 1 | 1 | 1 | 0 | 0 | 1.0 | 0 | 1.0 | 1 | 1.0 | 0 | 0 | 1.0 | 0 | 0 |
| Score(%) | 84.4 | 40.6 | 82.1 | 28.6 | 56.2 | 68.8 | 32.1 | 60 | 46.4 | 21.9 | 46.4 | 25 | 40 | 32.1 | 35.7 |
